# Tilapia as a model fish for biomonitoring of metal pollution in dams associated with mining watersheds: contrasting diagnosis from different tissues and health risk assessment

**DOI:** 10.1007/s10653-024-02232-8

**Published:** 2024-09-24

**Authors:** Federico Páez-Osuna, Aldivar Castro Espinoza, Eduardo Tirado Figueroa, César J. Saucedo Barrón, Magdalena E. Bergés-Tiznado

**Affiliations:** 1 Instituto de Ciencias del Mar y Limnología, Unidad Académica Mazatlán, Universidad Nacional Autónoma de México, Joel Montes Camarena S/N, P.O. Box 811, 82000 Mazatlán, Sinaloa Mexico; 2 Miembro de El Colegio de Sinaloa, Antonio Rosales 435 Pte., Culiacán, Sinaloa Mexico; 3 Secretaría de Agricultura, Ganadería y Pesca, Gobierno del Estado de Sinaloa, Instituto Sinaloense de Acuacultura y Pesca, Fray Servando Teresa de Mier 1870, 80129 Culiacán, Sinaloa Mexico; 4 Universidad Politécnica de Sinaloa, Carretera Municipal Libre Mazatlán Higueras Km 3, 82199 Mazatlán, Sinaloa Mexico; 5 Posgrado en Ciencias del Mar y Limnología, Universidad Nacional Autónoma de México, P.O. Box 811, 82000 Mazatlán, Sinaloa Mexico

**Keywords:** Cadmium, Copper, Zinc, Lead, *Oreochromis aureus*, Gulf of California

## Abstract

Tilapia is a model fish species used as a pollution biomonitor due to its tolerance and availability in many contaminated sites. Blue tilapia *Oreochromis aureus* specimens (n = 320) were collected in eleven dams influenced by mining in the SE Gulf of California region (dams 1, 2 and, 3 comprise 55 mining sites; dam 4 comprises 8; dams 6, 8, 10, and 11, ≤ 6; and dams 5, 7, and 9 include 19, 20, and 16 mining sites, respectively). Cadmium, Cu, Pb, and Zn concentrations were analyzed in the muscle, liver, gills, and guts to identify metal pollution and evaluate risks and seasonal changes. The distinct tissues exhibited different metal accumulation capacities, therefore allowed develop a diagnosis comparative between the eleven dams. In general, metal concentrations were higher in dams 1, 2, 5, and 9, which are associated with more mining sites in their sub-basins. The four metals exhibited the highest levels in the tilapia liver in dams 1 and 2, which can be related to the present and past mining activity in the lower watershed (55 sites) and the geothermal activity in these dams. In general, Zn exhibited the highest level in the tilapia livers from dams 1, 2, 3, 4, 5, and 10 compared to the maximum mean (220 µg/g) concentrations previously recorded. The non-carcinogenic risks indicated that the Pb risk was enhanced when the intake was ≥ 231.5 g week^−1^ of tilapia muscle, indicating a potential risk of adverse health effects for the entire population.

**Supplementary Information:**

The online version contains supplementary material available at 10.1007/s10653-024-02232-8.

## Introduction

Metal pollution associated with incidents and uncontrolled mine drainage in streams and rivers is often related to mining activities (e.g., Páez-Osuna et al., 2017, 2023a, 2023b). Hence, past and present mining is among the most metal sources in aquatic and terrestrial ecosystems (Páez-Osuna et al., 2015, 2017, 2023a). The emission and discharge of enormous quantities of materials occur directly from milling plants or indirectly through accidental impoundment failures (Kossoff et al., 2014). Mining generates considerable waste volumes, in which stream tailings are the leading waste source deposited in impoundments behind dams. However, these dams frequently fail and liberate massive tailing quantities into river catchments. These failures are exacerbated during rainy seasons (Kossoff et al., 2014) or extreme rains due to the fluvial dispersion of pollutants from impoundments. Metals and metalloids are present in tailings and emissions from the mining activity since no extraction process reaches 100% efficiency. Generally, As, Cu, Cd, Hg, Pb, Se, and Zn, among other elements, display high concentrations (Kossoff et al., 2014). Thus, mining areas are frequently regarded as one of the most contaminated locations for metals. Moreover, increasing economic activities, such as agriculture, livestock, and industry, have emerged in ancient mining areas (Zhu et al., 2018).

In Mexico, the mining industry is one of the most traditional economic activities primarily metalliferous, dedicated to producing Ag, Au, Cu, Fe, and Zn (Páez-Osuna et al., 2017, 2023a). In the lower watersheds of the states of Sonora, Sinaloa, Chihuahua, and Durango, the continental margin of the Gulf of California, there are ~ 225 geological and mining interest sites that were or are being exploited for Ag, Au, Cu, Fe, Mo, barite, halite, gypsum, and opal production (INEGI, 2016a, 2016b). Under such a scenario, coupled with the absence of an efficient strategy to follow up on mining activity regulations, a high incidence of spills has been evidenced in the Gulf of California eco-region. At least nine large-magnitude accidents (10,800–300,000 m^3^) occurred between 2013 and 2022 (Páez-Osuna et al., 2017, 2023a), in which most originated on the continental margin of the Gulf of California (NW Mexico). In addition, minor mining spills associated with commercial and artisanal mining occur, but these are not documented.

Metal accumulation in fish can vary according to the degree of exposure, and water parameters, including pH, hardness, organic matter, temperature, and salinity. In addition, metal levels can also vary according to the biological characteristics of fish, such as age, sex, length, weight, feeding habits, habitat, and metabolic status (Ali & Khan, 2018). Nevertheless, metal bioaccumulation in fish is initiated mainly by uptake via the food chain and direct exposure to metals present in the water and sediments (Sheikhzadeh & Hamidian, 2021). Therefore, environmental concentrations of metals in the habitat determine uptake levels through these three routes. Consequently, the metal levels in fish tissues could reveal the health status of the water bodies where they inhabit, including rivers, dams, estuaries, or coastal lagoons.

Regarding metal bioaccumulation, different fish tissues have diverse accumulation capacities depending on their structure and functions. Metabolically active fish tissues, such as the liver and kidney, may accumulate higher metal levels compared to other tissues, such as the muscle (Bergés-Tiznado et al., 2015, 2019; Páez-Osuna et al., 2023a, 2023b). Higher metal concentrations in the liver compared to those in muscle have been reported in both marine (Páez-Osuna et al., 2017) and freshwater fish species (Páez-Osuna et al., 2023a, 2023b). Thus, metal concentration in fish gills indicates metal concentrations in the ambient water, while those in the liver indicate long-term metal accumulation (Pandey et al., 2017). Conversely, fish meat (muscle) is crucial in environmental biomonitoring because the human population highly consumes this tissue. Consequently, metal accumulation in fish muscle from the viewpoint of human health is of significant concern. Therefore, it should be evaluated, especially in regions located in mining watersheds and where activities contribute to loading metals. Considering this behavior, various fish species have been used for the environmental biomonitoring of metal pollution. Metals may compromise the health benefits of fish consumption, which can adversely affect humans if they consume these elements at toxic levels. Therefore, monitoring metal concentrations in fish meat (muscle) is essential to ensure compliance with food safety regulations and consumer protection (Bosch et al., 2016). Additionally, the importance of fish as biomonitors of metal pollution and their usefulness in forensic studies for tracing pollution sources has been proposed (George et al., 2012).

The tilapia is a model fish species frequently used as a biomonitor of water pollution due to its tolerance and availability in many contaminated sites (*e.g.,* Shek & Chan, 2015; Ndimele et al., 2017; Páez-Osuna et al., 2023a, 2023b). This species is suitable due to its ability to accumulate metals, its sensitive response to pollutants, and its global distribution in inland and estuarine waters (Stickney, 2017). Tilapia is extensively reared in ponds, reservoirs, and dams. In addition, it can tolerate poor water quality and resist viral, bacterial, and parasitic diseases. This fish is, therefore, a model pollutant-resistant species for the water pollution biomonitoring (Shek & Chan, 2015).

The tilapia is the fourth largest species group in global aquaculture production (FAO, 2021), and also, is the most utilized fish in aquaponics systems coupled with vegetables and aromatic herbs (Yep & Zheng, 2019). In Mexico, tilapia is the second most important aquaculture group, with 53,000 t of production in 2018. Mexico is the second most important country in aquaculture tilapia fisheries, with 116,000 t registered in 2018. Mexico is also the second largest international market for tilapia products, with ~ 228,000 t imported in 2018 (FAO, 2021). However, metal pollution in tilapia has been poorly documented despite the importance of freshwater fish as a protein source for local diets.

This study aimed to determine Cd, Cu, Pb, and Zn concentrations in the muscle, gills, liver, and guts of *O. aureus* specimens collected from eleven dams located in NW Mexico, where tilapia is introduced and bred. Also, to assess the potential human health risk via fish consumption by comparing metal levels in the muscle with Mexican and international permissible limits for human consumption. The implicit hypothesis is that metal concentrations in the liver of tilapia associated with the bioavailability of such metals throughout its life in the dams can evidence metal pollution each dam´s watershed, where mining was or is practiced.

## Materials and methods

### Study area

**Fig. 1:**
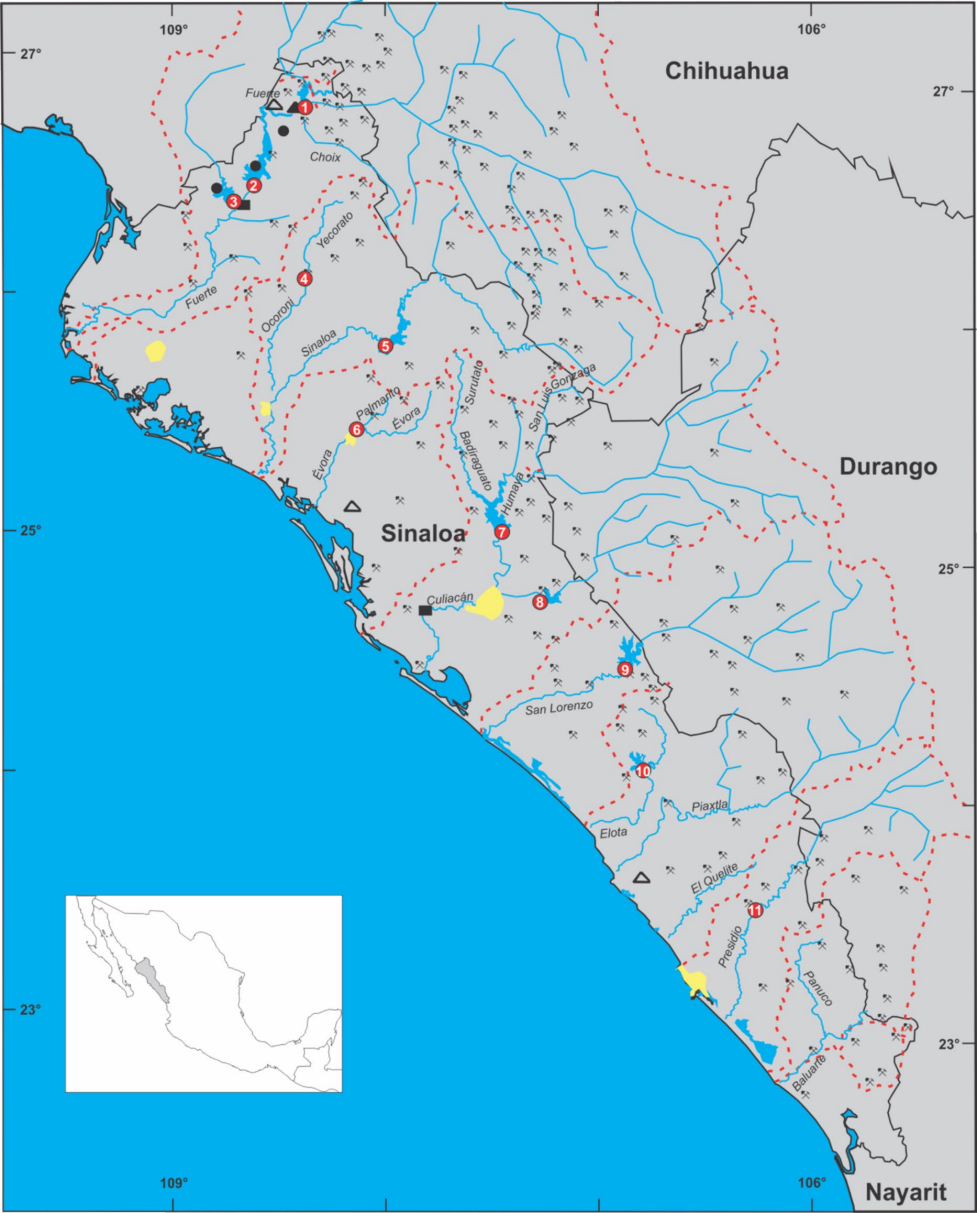
Map showing Sinaloa state, SE Gulf of California (NW Mexico), including the position of the dams (numbers in red circle), mining sites (hammer and pick, mining symbol), and geothermal activity (black circles). Discontinue red lines refer to the limits of the lower watershed of each river, and the yellow spots refer to the main cities

The study area includes a coastal plain with eleven rivers and 242 streams in the SE Gulf of California region (Fig. 1), transporting about 15,200 million m^3^ of water annually. A hydraulic infrastructure was developed to take advantage of such drainage, which includes irrigation districts and eleven dams that provide this region with 20,319.5 million m^3^ of water storage capacity (Beltrán-Álvarez et al., 2015). Sinaloa has a population of ~ 3.1 million people and a vast agricultural area (~ 1.1 million ha). The economic value of their products (~ 11.8 million tm per year) represents ~ 9.6% of the total agriculture production value in Mexico (CODESIN, 2021). Therefore, establishing the environmental status of dam waters is essential for the agricultural sector and all other activities that depend on water resources, such as aquaculture and fishing in the dams, livestock, tourism, industry, and especially human health.

Figure 1 shows the location of the eleven dams constructed in Sinaloa essentially to have water throughout the year and allow the development of agricultural activities, to generate electricity, and prevent flooding. The development of fishing and recreation was proposed as a secondary use. Table 1 summarizes the general characteristics of the eleven dams: the number of fishermen that operate in the eleven dams is ~ 2000, and the annual production can reach ~ 12,000 t. The number of mining sites in the sub-basins of the eleven dams is 139. The great agricultural activity occurs in the lower basins near the coastal plain, several km from the dams. Three fish species sustain the fishing activity in the dams: *Cyprinus carpio*, *Micropterus salmoides,* and tilapia *O. aureus;* this last fish species is the most important. Over the previous two decades, fish production has significantly changed due to weather, water quality, fishing activity, and the number of introduced fish hatchlings yearly (Beltrán-Álvarez et al., 2015).

**Table 1:** General characteristics of the dams studied in the SE Gulf of California

Official/common name of dam (location number in Fig. 1)	River	Area (ha)	Capacity (× 10^6^ m^3^)	Water temperature (°C)	Fishermen (number)	Annual production (t)^a^	Number of mining sites^b^
Luis Donaldo Colosio/Huites (1)	El Fuerte	9317	4568	16–31	274	38–400	55
Miguel Hidalgo C./El Mahone (2)	El Fuerte	12,200	3600	16–29	244	236–2000	55
Josefa Ortiz D./ El Sabino (3)	Alamos	5200	607	18–29	261	18–1940	55
Guillermo Blake/ El Sabinal (4)	Ocoroni	2700	350	–	–	–	8
Gustavo Díaz O./ Bacurato (5)	Sinaloa	7000	2900	18–32	136	450–1400	19
Eustaquio Buelna/ Guamúchil (6)	Mocorito	4700	344	20–32	67	5–200	5
Adolfo López M./ El Varejonal (7)	Humaya	11,300	3160	19–31	229	100–1200	20
Sanalona (8)	Tamazula	4500	845	20–31	103	40–900	5
José López P./ El Comedero (9)	San Lorenzo	9200	3400	21–31	197	< 100–1100	16
Aurelio Benassini V./ El Salto (10)	Elota	3200	410	21–32	203	300–1800	5
Picachos (11)	Presidio	2100	328	21–31	214	289–864	6
Total		71,417	20,512		1928	1575–11,804	139

^a^Range of production during the last 10 years; ^b^number of mining sites in the sub-basin (Data from Beltrán-Álvarez et al. 2015; INEGI, 2016a, 2016b)

### Sampling and metal analyses

Three hundred and twenty tilapia specimens were collected in the eleven dams (Fig. 1, Table 1). Samples were obtained during the rainy (August–October 2015) and dry seasons (February–March 2016). The individuals sampled were identified (Froese & Pauly, 2022) as blue tilapia *O. aureus*. A set of 12–15 fish specimens was collected from each dam (Table 3SM1) by local fishermen using cast nets. After taxonomic and sex identification, each specimen was measured (total length), weighed, and dissected to separate the liver, gills, guts, and a portion of the dorsal muscle. Tissue samples were kept frozen until laboratory processing and metal analysis.

The analytical procedure was previously described elsewhere (Páez-Osuna et al., 2023a, 2023b), briefly, lyophilized tissues of each specimen were digested with nitric acid (5 mL of concentrated ~ 70% Instra-analyzed J.T. Baker) of duplicate aliquots (~ 300 mg dry tissue) and carried out using Teflon vials (Savillex) at 125 °C for 3 h. Only the livers were digested using H_2_O_2_ (2 mL concentrated at 30%) and nitric acid (3 mL). Trace metals were quantified by atomic absorption spectrophotometry: Cd, Pb, Cu (graphite furnace), and Zn (flame). Elemental concentrations were expressed as μg g^−1^ on a wet weight basis (ww). Blanks and DORM-4 (fish protein) standard reference material were digested (one for each 15 sample batch) using the same procedure to evaluate accuracy and precision. Measured concentrations in reference material (NRC-CNRC, 2012) were within certified intervals; recoveries were 101.7 for Cd, 93.1 for Pb, 85.3 for Cu, and 103.0% for Zn (Table 1SM).

### Risk assessment

The non-cancer risk assessment was calculated as the individual target hazard quotient (THQ) and the sum of THQs as the hazard index (HI) by comparing the estimate of exposure to a reference dose (RfD) for oral exposures (EPA, 2005a): THQ = [EF × ED × FIR × C/RfD × BW × AT] × 10^–3^ and HI = ΣTHQ. Where EF is an exposure frequency of 365 days/year, ED is a 70-year exposure period, C is the mean concentration of the element (mg/kg), BW is the population body weight (75, 65, 50, and 20 kg for adult men, women, teenagers (12–17 years old), and children (3–5 years old), respectively), AT is the average exposure of 25,500 days, and FIR is the food ingestion rate under two different consumption scenarios. The first according to the specific fish species annual intake for Mexico (tilapia = 2.08 kg per capita), and the second under an intake ration equal to the total fish consumption rate (12.07 kg) per capita in 2021 (CONAPESCA, 2022). The FIR considered for each scenario was 39.9 g week^−1^ of tilapia (5.7 g day^−1^; FIR 1), and the second consumption scenario was 231.5 g week^−1^ (33.1 g day^−1^; FIR 2). There will be a risk if THQ or HI > 1. Furthermore, the RfD data for Cd (0.001 mg kg^−1^ BWday^−1^) and Zn (0.3 mg kg^−1^ BW day^−1^) were obtained from the IRIS Assessment Base (EPA, 2023). It is important to notice that Pb RfD has been reviewed, but the value is not estimated; also, Cu has not been evaluated. Lead, and Cd have been classified as probable human carcinogens based on sufficient evidence of animal carcinogenicity evidence (EPA, 2023; OEHHA, 2023); cancer risk (CR) was also evaluated as CR = CDI × SF, where CDI is the product of the average element concentration and the intake of the two specific scenarios mentioned before, divided by the corporal BW. Cd and Pb´s slope factor (SF) were 1.5 mg kg^−1^ BW day^−1^ and 0.0085 mg kgBW day^−1^, respectively (OEHHA, 2023).

Finally, a safe intake was calculated according to the Provisional Tolerable Intake (PTI) per BW set by the Joint FAO/WHO Expert Committee on Food Additives (JECFA). The data for each element were (WHO, 2021): Cd 25 μg kg^−1^ BW month^−1^; Cu 0.5 mg kg^−1^ BW day^−1^; and Zn 0.3 mg kg^−1^ BW day^−1^. The PTI for Pb was withdrawn as it is no longer considered protective, but the last estimated exposure for adults and children (under four years old) were 0.02–3 and 0.03–9 μg kgBW day^−1^, respectively. The Pb data used in this study for PTI and RfD was 0.01 μg kgBW day^−1^ to establish a better approach to avoid risks.

### Statistical analysis

The databases were analyzed in Excel for exploratory analysis, and the variables were tested using STATISTICA (version 7, StatSoft Inc.). Data were tested for normality (Kolmogorov–Smirnov and Lilliefors tests); as they were not normal, the metal datasets were statistically compared with a Kruskal–Wallis nonparametric ANOVA among elements and tissues. The influence of sex was compared with a Mann–Whitney U test; Spearman rank correlations (R) were used to determine associations among metal levels, length, and weight.

## Results

**Fig. 2:**
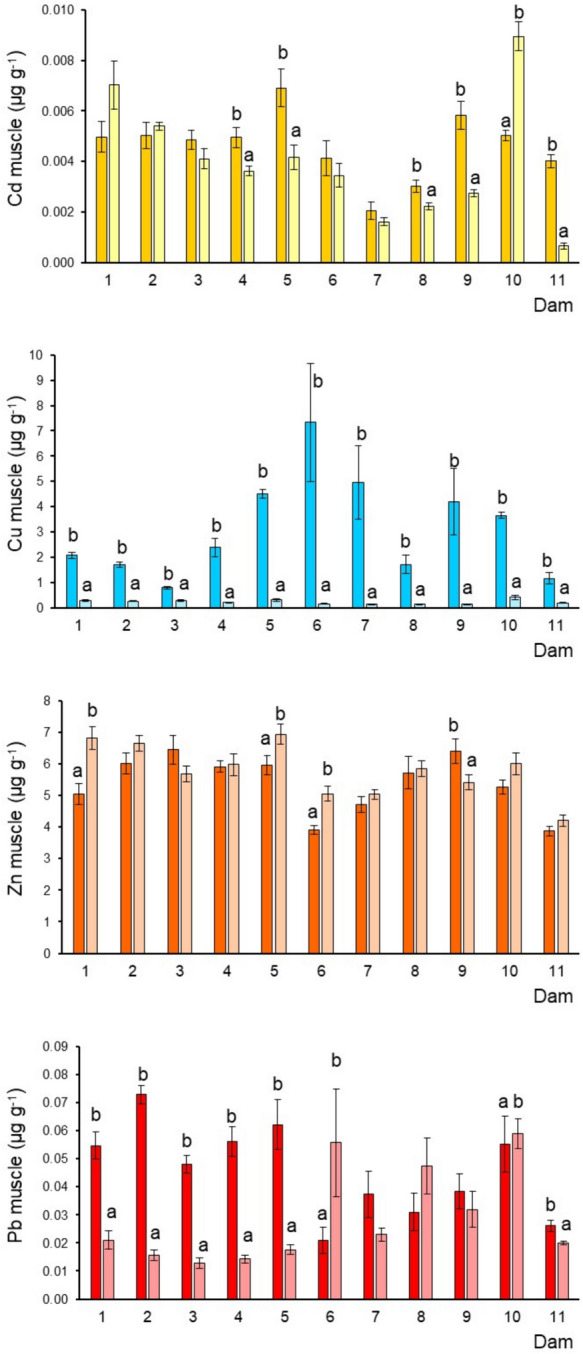
Cadmium, Cu, Zn, and Pb (mean ± SE, ww) concentrations in the tilapia muscle from each dam by sampled season: deep and soft color correspond to dry and rainy season; different letters indicate significantly different (*p* < 0.05) mean concentrations between seasons in each dam; SE = standard error

**Fig. 3:**
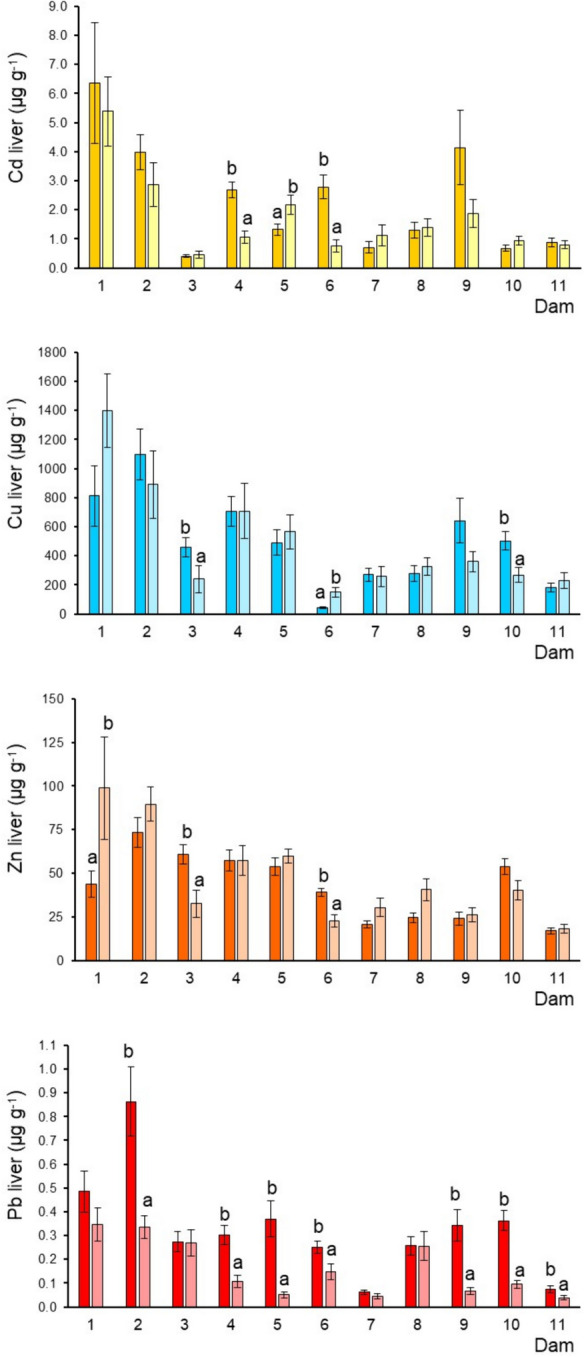
Cadmium, Cu, Zn, and Pb (mean ± SE, ww) concentrations in tilapia liver from each dam by sampled season: deep and soft color correspond to dry and rainy season; different letters indicate significantly different (*p* < 0.05) mean concentrations between seasons in each dam; SE = standard error

**Fig. 4:**
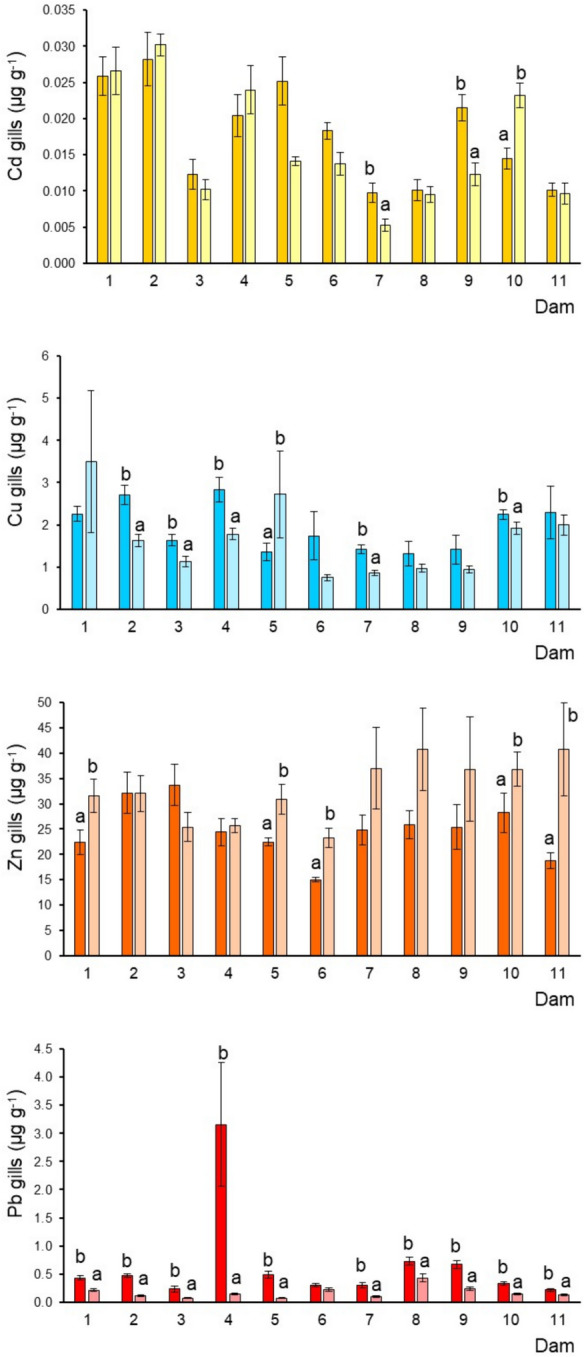
Cadmium, Cu, Zn, and Pb (mean ± SE, ww) concentrations in tilapia gills from each dam by sampled season: deep and soft color correspond to dry and rainy season; different letters indicate significantly different (*p* < 0.05) mean concentrations between seasons in each dam; SE = standard error

**Fig. 5:**
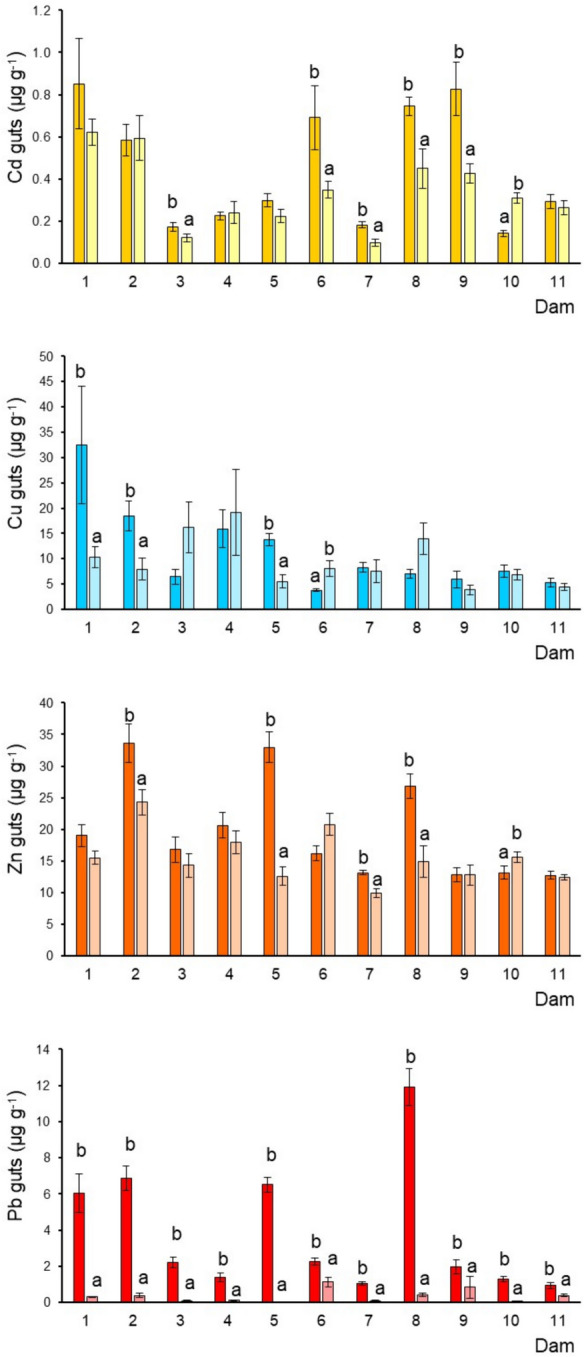
Cadmium, Cu, Zn, and Pb (mean ± SE, ww) concentrations in tilapia guts from each dam by sampled season: deep and soft color correspond to dry and rainy season; different letters indicate significantly different (*p* < 0.05) mean concentrations between seasons in each dam; SE = standard error

The smallest tilapia were observed in dams 2 and 6, while the largest were in dams 10 and 11 (Table 3SM1) during the dry and rainy seasons. The biometric data of the studied fish (female mean, 19.0–32.9 cm; male mean, 19.8–36.9 cm) indicated that the specimens were adults since most were above the estimated sexual maturity size (13–20 cm) (Froese & Pauly, 2022). In general, the metal concentration sequence differed among tissues and dams (Figs. 2, 3, 4 and 5). In the eleven dams, the sequence was consistent in the liver Cu > Zn > Cd > Pb while in muscle, gills, and guts the tendency was Zn > Cu > Pb > Cd. Cu and Zn had the highest levels, while Pb and Cd had the lowest. Metal concentrations showed significant variability in the tissues, particularly in the liver and, most significantly in the guts, between tilapias of different dams. Such variability can be caused by different habitat conditions, as well as by the variable exposure associated with each habitat occupied by tilapia in each dam; oxygen levels, redox, organic matter composition, pH, alkalinity, and temperature are factors that directly influence chemical speciation and bioavailability. Metals generally accumulate in the liver, followed by the guts, gills, and muscle. Differences in element accumulation in tissues are mainly due to each metal´s biochemical characteristics, biological function, and bioavailability.

### Metals in tilapia muscle (accumulation of human concern)

Fish muscle (meat) is key and of major concern in environmental biomonitoring because the human population consumes this tissue. Cadmium concentrations were higher during the dry season in dams 3, 4, 5, 6, 7, 8, 9, and 11, although the highest levels were observed during the rainy season in dams 10 and 1 (Fig. 2), characterized by a low (5) and high (55) presence of mining sites, respectively (Table 1). The lowest Cd level was found in dam 11, during the rainy season (six mining sites), and dam 7 (20 mining sites) in both seasons. Copper exhibited a defined pattern with the highest concentrations in the dry season in the dam 6 and the lowest in dams 3 and 11 associated with 55 and six mining sites in their lower basins (Table 1). Contrarily to Cu, Zn exhibited a pattern with comparable concentrations in both seasons. However, slightly higher levels were observed during the rainy season (Fig. 2). Dams 5 and 1 exhibited the highest Zn levels, while dam 11 showed the lowest (Fig. 2). For Pb, most dams showed a higher concentration in muscle during the dry season, highlighting dam 2 with the highest level, and dam 11 with the lowest (Fig. 2).

### Metals in the tilapia liver (long-term exposure to metals)

The four metals exhibited the highest concentrations in dam 1, followed by dam 2, while the lowest in dam 3 for Cd, dam 6 for Cu, dam 11 for Zn, and dam 7 and 8 for Pb (Fig. 3). Cadmium showed significant differences between the seasonal means only in dams 4, 5, and 6, with an inconsistent pattern. Copper and Zn also showed an inconsistent pattern and high seasonal variability, while Pb tended to show higher levels during the dry season, which was more notorious in dam 2 (Fig. 3). Dams 1 and 2 are associated with the most mining sites (55), while dams 6 and 11 have less (5 and 6, respectively) (Table 1). Another factor that could influence the metal concentrations in the tilapia livers is geothermal activity, which is present in dams 1, 2, and 3 (INEGI, 2016a, 2016b, 2016c). However, dam 3 showed moderate Cu, Zn, and Pb levels and the lowest Cd level. Dam 9, which had a mining spill in 2013, showed moderate to high levels of the four metals (Fig. 3), indicating that the reservoir could have naturally depurated during 2013–2017.

### Metals in the tilapia gills (metals in the ambient water)

Cadmium concentrations exhibited high levels in dams 1 and 2 (both seasons), 5 (dry season), and 10 (rainy season) (Fig. 4). While the lowest concentrations were observed in dams 7, 8, and 11. Copper did not show a defined pattern due the high seasonal variation. Nonetheless, dams 1 and 5 showed the highest levels in the rainy season, while dam 6 (rainy season) showed the lowest (Fig. 4). In general, Zn concentrations were higher in the rainy season, and the highest levels were found in dams 11, 8, and 7. The Pb pattern was well defined with high concentrations in the dry season, and the highest level was observed in dam 4, while the lowest concentration was evidenced in dams 11, 3, and 7.

### Metals in the guts (tilapia diet)

The guts and their content reflect the metal concentration related to the recent food uptake during the last hours before specimens were collected. Tilapia is an omnivore that eventually captures its food (detritus) from the sediments (Stickney, 2017). The Cd concentration in guts was more elevated in dams 1 and 9 during the dry season. While generally, the lowest levels in dams 3 and 7 were observed during the rainy season (Fig. 5). Copper exhibited a similar pattern, with the highest concentrations observed during the dry season in dams 1 and 2 and the lowest during the rainy season in dams 11 and 9. Zinc showed the highest levels in most dams during the dry season, notoriously in dams 2 and 5 and the lowest in dams 11 and 7. Lead presented a well-defined pattern, with higher concentrations in dams 8 and 2 during the dry season. While the lowest Pb levels were evidenced during the rainy season in dams 10, 5, 4, 3, and 7 (Fig. 5).

### Health risk assessment

Generally, this study found that metal levels in the tilapia muscle from the eleven dams did not exceed the maximum permissible limits established by various national or international organizations. These limits are as follows: the Official Mexican Standard 0.5 µg/g ww [3.0 µg/g dw] for Cd (DOF, 2011); the European Union 0.3 µg/g ww [1.8 µg/g dw] for Cd (CREC, 2006); the U.S. Food and Drug Administration 0.5 µg/g ww for Cd [3.0 µg/g dw] and 1.3 µg/g ww for Pb [7.74 µg/g dw] (FDA, 1993a, 1993b); Australia 10 µg/g ww [60 µg/g dw] for Cu and 150 µg/g ww [900 µg/g dw] for Zn (Nauen, 1983); and India 10 µg/g ww [60 µg/g dw] for Cu and 50 µg/g ww [300 µg/g dw] for Zn (Nauen, 1983).

Table 2 summarizes the results on the THQ, HI, and CR values for two consumption scenarios for different population strata: as men and women (> 18 years old nor adults), teenagers (12–17 years old), and children (2–5 years old). In general, it is observed that THQ values were < 1.0; only in the case of children the THQ_Pb_ showed values > 1.0, and for all strata (men THQ_Pb_ = 1.67; women THQ_Pb_ = 1.93; teenager THQ_Pb_ = 2.51; and children THQ_Pb_ = 6.27) when consume is 231.5 g week^−1^. These results are reflected in the HI, which reaches the highest value in the children (HI = 6.31) when consumption of tilapia is high. Similar to the THQ values, CR values exhibited the highest values (1.1–4.3 × 10^–6^) in the four strata considered for Cd when consumption of tilapia is 231.5 g week^−1^.

**Table 2:** Risk assessment (THQ, HI and CR) with two consumption scenarios for different population strata as men and women (> 18 years old nor adults), teenagers (12 – 17 years old) and children (2–5 years old)

Risk Index	FIR1 (39.9 g week^−1^)				FIR2 (231.5 g week^−1^)			
	Men	Women	Teenagers	Children	Men	Women	Teenager	Children
THQ_Cd_	< 0.01	< 0.01	< 0.01	< 0.01	< 0.01	< 0.01	< 0.01	0.01
THQ_Pb_	0.29	0.33	0.43	1.08	1.67	**1**.93	2.51	6.27
THQ_Zn_	< 0.01	< 0.01	< 0.01	0.01	0.01	0.01	0.01	0.03
HI	0.29	0.33	0.44	1.09	1.68	1.94	2.53	6.31
CR_Cd_	5.0 × 10^–7^	5.7 × 10^–7^	7.5 × 10^–7^		2.9 × 10^–6^	3.3 × 10^–6^	4.3 × 10^–6^	1.1 × 10^–6^
CR_Pb_	2.4 × 10^–8^	2.8 × 10^–8^	3.7 × 10^–8^	9.2 × 10^–8^	1.4 × 10^–7^	1.6 × 10^–7^	2.1 × 10^–7^	5.3 × 10^–7^

Men = 75 kg BW; women = 65 kg BW; teenagers = 50 kg BW; children = 20 kg BW; FIR 1 = 2.08 kg annual per capita of tilapia consumption and FIR 2 = 12.07 kg annual per capita fish intake in Mexico (CONAPESCA, 2021). Numbers in bold mean THQ or HI > 1

## Discussion

### Metals in fish tissues

Overall, the metal concentrations were higher in dams 1, 2, 5, and 9 and lower in dam 11. The highest concentrations in the muscle were measured in dams 10, 1, and 5 for Cd, 6 and 5 for Cu, 5, 1, and 2 for Zn, and 6 and 5 for Pb. For the liver, the highest levels were found in dams 1, 2, and 9 for Cd and 1 and 2 for Cu, Zn, and Pb. High levels in the gills were evidenced in dams 1 and 2 for Cd and Cu, in 8 and 11 for Zn, and 4 for Pb. In guts, the higher concentrations were quantified in dams 1, 9, and 8 for Cd, 1 and 2 for Cu, 1 and 5 for Zn, and dams 5 and 8 for Pb. Dam 11 exhibited the lowest levels of the four metals in muscle, Zn in the liver, Cd and Pb in the gills, and Cu and Pb in the guts.

The four metals showed the highest concentrations in dam 1, followed by dam 2, while the lowest in dam 3 for Cd, dam 6 for Cu, dam 11 for Zn, and dam 7 and 8 for Pb (Fig. 3). This behavior can be related to the present and past mining activity. During the routine operation of mining activity, pollution is one of the most common sources of highly toxic chemicals in aquatic and terrestrial ecosystems (Mapenzi et al., 2020). Large quantities of materials are discharged either directly from milling plants or indirectly through accidental impoundment failures. The mining industry produces enormous volumes of waste, mainly tailings, which are often stored in impoundments behind dams. These can fail and have subsequent environmental, economic, and human health impacts (Kossoff et al., 2014). Metals and metalloids are present in tailings since no extraction process reaches complete efficiency, and Cu, Cd, Hg, and Zn are usually present in elevated concentrations (Páez-Osuna et al., 2023a).

The general pattern of higher metal levels in the liver compared to the other tissues coincides with previous studies in freshwater (Páez-Osuna et al., 2023a, 2023b; Vasco-Viteri et al., 2023; Yap et al., 2015) and marine (Páez-Osuna et al., 2017; Ruelas-Inzunza et al., 2020) fish species. The high and consistent accumulation of Cd and Cu in all dams, and partially (except dams 7, 8, and 11) Zn in the liver, is directly associated with metabolism and respiration (Páez-Osuna et al., 2023b) since diet and water are the main capture and assimilation routes. The liver has functional groups formed by sulfhydryl (–SH), carboxyl (–COOH), and amino (–NH_2_) with a strong affinity for these metals, so these could be bound and retained (Moreno-Sierra et al., 2016). The liver metabolizes substances that come through the blood (Torres et al., 2010). Moreover, this organ synthesizes metallothioneins which interacting with Cd, Cu, and other metals reducing their toxicity (Yap et al., 2015). Metallothionein induction in fish is high in metal uptake, storage, and excretion tissues, such as the liver and kidney (Páez-Osuna et al., 2017, 2023a, 2023b; Viarengo et al., 2007). The liver is a highly active organ involved in the storage and detoxification of metals in fish, particularly Cd and Cu. Therefore, it has been considered a biomonitor of metal pollution, given that liver concentrations are proportional to those in the environment; Yap et al. (2015) observed a limited proportionality with sediments in contaminated and uncontaminated ponds, and Páez-Osuna et al. (2023a) related the mortality and high metal concentrations in tilapia with the levels and speciation of metals in the water.

The lower levels of Zn, Cu, Cd, and Pb in muscle tissue are related to the proportion of muscle to the fish body mass; this may act as a terminal reservoir that progressively accumulates metals from other organs (Liu et al., 2014). In addition, this tissue may reflect low metallothioneins levels (Allen-Gil & Martynov, 1995). Tilapia is an omnivore fish that consumes detritus from the sediments (Stickney, 2017). Once ingested, the metal uptake occurs in the intestines through membranes via transporter proteins and ionic channels. After reaching the liver, metals are released into the general blood circulation and finally reach secondary accumulation tissues, such as the muscle (Le Croizier et al., 2018).

### Metal variability with body size

In determined cases, the morphometric variables total length (TL) and weight, in tilapia were significantly correlated (*p* < 0.05) with the measured metals in the studied tissues (Tables 3SM2-3SM12 and Figs. 3SM1-3SM23). Some correlation examples are shown in Fig. 3SM24. The number of significant (*p* < 0.05) correlations differed among the dams and metals; dam 7 had the most while dams 10 and 11 had the least cases. Overall, 41 significant correlations were observed between metal concentrations and size (TL) (Tables 3SM2-3SM12) in the present study. Dams 9, 10, and 11 did not exhibit significant (*p* > 0.05) correlations, while dams 2 and 7 exhibited seven significant (*p* < 0.05) correlations each. The tissue that showed a greater number of correlations in the dams was muscle (15) and guts (11), and the highest number of correlations concerned Pb (17) and Cu (12). Metabolic processes in the fish body generally regulate essential metals, so in many cases, they do not correlate with length or weight (Kojadinovic et al., 2007; Moreno-Sierra et al., 2016). Furthermore, these discrepancies may be related to size and weight differences of the specimens analyzed in each reservoir.

These correlation graphics showed that the sex of tilapias does not significantly influence the metal concentration of the studied tissues. When tilapias were larger, they tended to consume a more concentrated diet (gut content) of Cu (Fig. 3SM1) and Pb (Fig. 3SM2) as was observed in dam 1 (Fig. 3SM1), Pb in dam 2 (Fig. 3SM5), Cu in dam 4, Cd (Fig. 3SM15), Pb (Fig. 3SM16), Cu (Fig. 3SM17) and Zn (Fig. 3SM18) in dam 7, and Pb in dam 8. This tendency observed in various dams partly explains the concentration variation of these metals with body size in certain tissues. Although the existence of size and dependent metal accumulation by aquatic biota is well recognized from classical studies, this pattern is difficult to explain since body size influence may be a function of any one or several age-dependent parameters. It may also be related to differences between the surface/volume ratio, as well as the metabolic and feeding rates of larger (older) and smaller (younger) individuals (Páez-Osuna et al., 1995, 2023a).

### Seasonal variation of metals

This study revealed differences in metal concentrations in fish tissues across all dams under investigation. Seasonal fluctuations toward metal accumulation in the fish were also recorded (Figs. 2, 3, 4, and 5). For instance, in muscle, higher concentrations of Cd were found during the dry season (dams 3, 4, 5, 6, 7, 8, 9, and 11) and for Cu (dam 6) (Table 1), while Zn exhibited a pattern with comparable concentrations in both seasons. In the liver, Cd showed significant differences between the seasonal means in dams 4, 5, and 6. Copper and Zn also showed an inconsistent pattern and high seasonal variability. At the same time, Pb tended to show higher levels during the dry season, which was more notorious in dam 2 (Fig. 3). In gills, Cd and Cu did not show a defined pattern due to the high seasonal variation. Nonetheless, Cu in dams 1 and 5 showed the highest levels in the rainy season. In general, Zn concentrations were higher in the rainy season. The Pb pattern was well defined with high concentrations in the dry season. Finally, in guts, the four metals exhibited a similar pattern, with the highest concentrations during the dry season. Temperature influences metal build-up, as a result of high temperature, more water passes through the gills, increasing the amount of metals taken up by the fish. Metal levels in the liver decrease during the winter (dry season) as a result of heightened metal absorption prompted by excretory organ clearance (Inayat et al., 2024). Most of the metals analyzed in the four tissues and the eleven dams presented the highest concentrations of these selected metals observed during the dry season (March and April), which contrasts with the found in various studies in which the maximum concentrations have been registered in summer (e.g., Hussain et al., 2021; Inayat et al., 2024). Rajeshkumar et al. (2018) observed that Cu, Cd, and Pb levels were significantly increased in different organs of the fish *Carassius carassius* in the winter and summer than in spring and autumn, which was related to a high influx of metals by the pollution from the surrounding industries. Lozano-Bilbao et al. (2020) related the high concentrations of Zn in the cold season of the fish *Trachurus picturatus* may be due to the effect that upwelling, while the summer are those that have the highest level of Cd and Pb, which are metals of anthropogenic character associated mainly with tourism. Although several studies have addressed metal bioaccumulation in fishes from different environments, the possible seasonal variations associated still need to be sufficiently examined.

### Comparison with other regions

Table 3 was elaborated to compare Cd, Cu, and Zn concentrations in tilapia muscle and liver found in this study with other reported worldwide; Pb was not included due to the limited number of studies. The highest levels of the three metals were found in the liver, whereas the lowest in the muscle, a pattern reported for an extensive spectrum of fish species (Páez-Osuna et al., 2017, 2023a, 2023b). The mean ranges reported for the tilapia liver from previous studies are 0.036–320 for Cd, 1.26–8758 for Cu, and 4.3–220 µg/g (dw) for Zn (Table 3). While in the dams of this study, the ranges of means were 2.15–30.2 for Cd, 574–5662 for Cu, and 89.2–418 µg/g (dw) for Zn, i.e., Cd and Cu were within such ranges, but Zn concentration was higher in the tilapia livers of this study. Notably, high levels were observed in dams 1, 2, 3, 4, 5, and 10, which exceed the maximum mean level previously reported (Table 3). This indicates that the tilapias from these reservoirs are long-term exposed to high bioavailable Zn concentrations in these dams, which can be related to the mining (Table 1) and geothermal activity in dams 1, 2, and 3 (INEGI, 2016a, 2016b, 2016c).

**Table 3:** Ranges and mean concentration (µg g^−1^ dw) of cadmium, copper, and zinc in tilapia worldwide

Species	Cd	Cu	Zn	Pollution	Region	Reference
*O. niloticus*				Municipal	Yaounde lake, Cameroon	Léopold et al. (2015)
Muscle	0.11–0.23	0.59–4.11	15.4–47.2			
*O. niloticus*				Wastewater ponds	Wetland, East Calcutta, India	Chatterjee et al. (2016)
Liver	–	320	315			
*O. niloticus*				Mining towns	Kafue River, Zambia	Mbewe et al. (2016)
Muscle	0.30	2.8	–			
Liver	2.0	49.5	–			
*O. niloticus*				Sewage urban and agriculture	Lake Phewam, Nepal	Rosseland et al. (2017)
Liver	1.3 (0.4–1.8)	660 (120–988)	97 (61–132)			
*O. niloticus*				Agriculture, industrial, and urbanism	Mariut and Edku lakes, Egypt	Abdel–Moneim et al. (2016)
Liver	0.036–0.205	1.26–3.29	4.3–23.4			
*O. niloticus*				Industrial	Ologe lagoon, Owo and Etegbin River, Nigeria	Ndimele et al. (2017)
Muscle	–	34.7	10.5			
*O. niloticus*				Mining area	La Angostura dam, Sonora, Mexico	Martínez–Durazo et al. (2021)
Muscle	–	35.5 ± 10.0	18.7 ± 6.5			
Liver	–	649 ± 298	51.7 ± 10.1			
*O. niloticus*				Mining area	El Cajon de Onapa dam, Sonora, Mexico	Martínez–Durazo et al. (2021)
Muscle	–	18.5 ± 1.4	37.5 ± 7.3			
Liver	–	660 ± 393	46.5 ± 17.6			
*O. niloticus*				Mining area	El Oviachic dam, Sonora, Mexico	Martínez–Durazo et al. (2021)
Muscle	–	20.3 ± 3.0	17.1 ± 6.9			
Liver	–	521 ± 232	89.3 ± 28.9			
*Tilapia zillii*				Agriculture, industrial	Cross River, SE Nigeria	Okogwu et al. (2019)
Muscle	–	1.50	20.0			
*O. esculentus*				Agriculture, mining	Rukwa lake, Tanzania	Mapenzi et al. (2020)
Muscle	–	0.25–1.52	64.0–133.5			
*O. mossambicus*				Mining activities	Yonki dam, Papua New Guinea	Kapia et al. (2016)
Muscle	< 0.01	2.64	–			
*O. mossambicus*				Ponds influenced by domestic effluents	Malaysia	Yap et al. (2015)
Muscle	0.34–0.84	1.4–2.1	15.7–25.6			
Liver	1.28–3.05	8.9–269	53.5–101.9			
*O. mossambicus*				Mining and agriculture	Aquaculture farms, Lhasa Tibet, China	Jiang et al. (2014)
Muscle	21.9	888	–			
Liver	320	4225	–			
*O. mossambicus*				Lagoons influenced by agriculture and livestock	Valley Culiacan, NW Mexico	Izaguirre–Fierro et al. (1992)
Muscle	0.4–0.6	4.6–6.0	14–19			
*O. aureus*				Mining area	El Salto dam, NW Mexico	Frías-Espericueta et al. (2010)
Muscle	0.28 ± 0.03	0.98 ± 0.53	12.1 ± 2.6			
Liver	0.71 ± 0.31	147 ± 67	38.9 ± 12.1			
*O. aureus*				Mining tailing spill	El Comedero dam, NW Mexico	Páez-Osuna et al., (2023a, 2023b)
Muscle	0.28 ± 0.06	1.48 ± 0.41	14.7 ± 1.0			
Liver	52.0 ± 24.6	8758 ± 3692	220 ± 50			
*O. aureus*				Mining	Dam 1, NW Mexico	This study
Muscle	0.036 ± 0.018	7.1 ± 0.6	35.1 ± 9.5			
Liver	30.2 ± 33.1	5662 ± 5072	364 ± 441			
*O. aureus*				Mining	Dam 2, NW Mexico	This study
Muscle	0.030 ± 0.012	5.9 ± 4.8	37.5 ± 7.1			
Liver	17.6 ± 13.6	5092 ± 4005	418 ± 18			
*O. aureus*				Mining	Dam 3, NW Mexico	This study
Muscle	0.024 ± 0.012	3.15 ± 1.78	36.3 ± 8.9			
Liver	2.15 ± 1.74	1790 ± 1682	239 ± 151			
*O. aureus*				Mining	Dam 4, NW Mexico	This study
Muscle	0.024 ± 0.006	7.74 ± 8.93	35.1 ± 5.9			
Liver	9.59 ± 6.41	3621 ± 2964	294 ± 143			
*O. aureus*				Mining	Dam 5, NW Mexico	This study
Muscle	0.036 ± 0.018	14.3 ± 13.1	38.1 ± 7.7			
Liver	8.97 ± 5.64	2703 ± 2020	292 ± 91			
*O. aureus*				Mining	Dam 6, NW Mexico	This study
Muscle	0.024 ± 0.012	16.1 ± 30.4	28.0 ± 5.4			
Liver	7.49 ± 6.87	574 ± 564	145 ± 73			
*O. aureus*				Mining	Dam 7, NW Mexico	This study
Muscle	0.012 ± 0.006	14.3 ± 25.6	29.2 ± 4.8			
Liver	4.77 ± 5.79	1349 ± 1144	132 ± 81			
*O. aureus*				Mining	Dam 8, NW Mexico	This study
Muscle	0.018 ± 0.006	5.4 ± 7.1	34.5 ± 8.3			
Liver	6.92 ± 5.44	1559 ± 1097	170 ± 105			
*O. aureus*				Mining	Dam 9, NW Mexico	This study
Muscle	0.024 ± 0.012	13.1 ± 24.4	35.1 ± 7.7			
Liver	15.4 ± 19.8	2303 ± 2441	128 ± 77			
*O. aureus*				Mining	Dam 10, NW Mexico	This study
Muscle	0.042 ± 0.018	11.9 ± 10.1	33.3 ± 7.1			
Liver	4.10 ± 2.72	1969 ± 1277	241 ± 105			
*O. aureus*				Mining	Dam 11, NW Mexico	This study
Muscle	0.018 ± 0.012	4.2 ± 4.8	23.8 ± 3.6			
Liver	4.31 ± 2.82	1036 ± 744	89.2 ± 37.9			

–, not analyzed; moisture levels considered to change from wet weight to dry weight, muscle 83.2%, and liver 80.5%

The Cd levels in the tilapia liver (2.15–30.2 µg/g dw) from the present study were relatively higher compared with those areas with no mining activity (Table 3), such as the Culiacan valley, Mexico (Izaguirre-Fierro et al., 1992), Malaysia (Yap et al., 2015), Mariut and Edku lakes, Egypt (Abdel-Moneim et al., 2016), and Lake Phewam, Nepal (Rosseland et al. (2017). The highest Cd levels are registered in the tilapia liver from Lhasa, Tibet, China, where mining and agriculture occur (Jiang et al., 2014), and in the blue tilapia *O. aureus* from dam 9 that in 2013 suffered a mortality event induced by a mining tailing spill (Páez-Osuna et al., 2015, 2023a). The Cu concentrations in the tilapia liver (574–5662 µg/g dw) in this work are also relatively high regarding those of mining and non-mining areas (Table 3), which are only lower than the tilapias sampled during a mortality event induced by a mining tailing spill (Páez-Osuna et al., 2023a). These results confirm that tilapias from dams 1, 2, 3, 4, and 5 have been exposed to Cu in the long-term, and those from dams 1 and 2 to Cd.

The Cd mean levels in the tilapia muscle (0.012–0.042 µg/g dw) from this study were within those reported elsewhere; they are lower than those from mining and non-mining areas (Table 3). On average, they are ten times below those measured in the same tilapia species sampled days after a mining spill event (Páez-Osuna et al., 2023a). The Cu level in the tilapia muscle (4.2–16.1 µg/g dw) was also similar to those registered worldwide, being lowest and comparable to those from non-mining and mining areas, respectively (Table 3). Furthermore, they are about two to ten times higher than those quantified during the mortality event induced by a mining tailing spill (Páez-Osuna et al., 2023a, 2023b). The Zn mean concentrations in the tilapia muscle (23.8–38.1 µg/g dw) from this study, were within levels from mining and non-mining areas (10.5–133 µg/g dw) (Tables 1 and 3). However, the first means are higher than those from the tilapia muscle (14.7 µg/g dw) measured during a mortality event induced by a mining tailing spill (Páez-Osuna et al., 2023a).

### Health risk assessment

Two direct human intake scenarios were considered with a FIR1 of 39.9 g week^−1^ (5.7 g day^−1^) and a FIR2 of 231.5 g week^−1^ (33.1 g day^−1^), which were the annual tilapia consumption per capita and the annual fish consumption in Mexico, respectively (CONAPESCA, 2022). The non-carcinogenic risks indicated that there is an evident risk (THQ and HI > 1) in children caused by Pb if the consumption scenario was 5.7 g day^−1^ of tilapia muscle (Table 2). The Pb risk was enhanced when the intake rose to scenario 2 (FIR2), given that THQ and HI > 1 for adults (men and women), teens, and children, meaning that all the population could be at risk of adverse health effects caused by Pb.

The estimated cancer risk probability was low (Table 2) for Cd and Pb. The FIR1 showed the highest probable CR by exposure to Cd of 1.9 × 10^–6^, meaning that 1.9 children over one million could probably get cancer caused by Cd. The CR by Pb exposure is lower than for Cd; the probabilities of CR in FIR2 were a slightly higher than in FIR1. The probabilities of developing cancer considered acceptable by the EPA (2005b) are from 10^–4^ to 10^–6^, and all CR values were < 2.0 × 10^–6^, meaning that the probability of developing cancer is < 2 in 1,000,000. This study´s CR probabilities were < 2 × 10^–6^, with low probabilities of cancer development by tilapia muscle consumption.

The maximum tolerable weekly intake must be very high to be at risk for the essential elements Cu (170.0 and 147.3 kg for adult men and women, 113.3 kg for teenagers, and 45.3 kg for children), but lower for Zn (27.9, 24.2, 18.6, and 7.4 kg for adult men and woman, teens, and children, respectively). The tolerable weekly intake for Cd was lower than Cu but higher than Zn (100.3, 86.9, 66.9 and 28.6 kg for adult men and women, teens, and children, respectively). Therefore, tilapia muscle consumption cultured in the 11 dams might be considered safe regarding the latter elements. However, the population might be at risk by Pb if the edible tilapia flesh is consumed weekly over 138.6 (men) and 120.2 g (women) for adults, 92.4 g for teenagers and 39.9 g for children. Lower portions must be consumed to avoid potential risks caused by Pb.

## Conclusions

The analyzed metals in this study were significantly (*p* < 0.05) correlated with tilapia body size (41 correlations between metals and TL). However, correlations were different between dams, tissues, and metals; the muscle and guts had more cases, and the mainly involved metals were Pb and Cu. In general, sex did not significantly influence the metal concentration of the studied tissues. Interestingly, larger tilapias tended to consume a more concentrated diet (gut content) of Cu, Zn, and Pb. This tendency observed partially in the dams explains the concentration variation of these metals with body size.

The Cd and Cu concentrations in the tilapia liver of this study were within the ranges reported previously in tilapias of other regions worldwide. In contrast, Zn exhibits a higher level in the tilapia livers, particularly in dams 1, 2, 3, 4, 5, and 10 (Fig. 1), which indicates that the tilapias from these reservoirs are long-term exposed to high bioavailable Zn. This can be attributed to the mining activity that occurs mainly in the basins of such dams and due to the geothermal activity in dams 1, 2, and 3. The adult men and women population must consume < 138.6 and 120.2 g, respectively; teenagers and children should consume less than 92.4 and 39.9 g of tilapia muscle to avoid adverse health effects caused by Pb. The probabilities of developing cancer due to Pb and Cd content in the edible flesh of tilapia were very low (< 1.9 × 10^–6^). The hypothesis proposed can be partially confirmed since metal concentrations in the tilapia liver evidenced metal pollution in the watershed of those dams with most mining sites. This was observed for Zn in dams 1, 2, 3, 4, 5, and 10. However, such pollution can also be associated with geothermal activity in dams 1, 2, and 3.

## Supplementary Information

Below is the link to the electronic supplementary material.

Supplementary file1 (DOCX 265 KB)

## Data Availability

The datasets used and/or analyzed during the current study are available from the corresponding author on reasonable request
