# Supplementary material for: Tilapia as a model fish for biomonitoring of metal pollution in dams associated with mining watersheds: contrasting diagnosis from different tissues and health risk assessment
Source: Environ Geochem Health. 2024 Sep 24;46(11):447. doi: 10.1007/s10653-024-02232-8 (PMC11422272; doi:10.1007/s10653-024-02232-8)
Supplement: Supplementary file 1 — Supplementary file1 (DOCX 265 KB) [file 10653_2024_2232_MOESM1_ESM.docx]

**Supplementary material (SM)**

Article title: **Tilapia as a model fish for biomonitoring of metal pollution in dams associated with mining watersheds: contrasting diagnosis from different tissues and health risk assessment**

Journal name: Environmental Geochemistry and Health

Author names:

Federico Páez-Osuna ^1,2,*^, Aldivar Castro Espinoza ^3^, Eduardo Tirado Figueroa ^3^, César J. Saucedo Barrón ^3^, Magdalena E. Bergés-Tiznado ^4, 5^

Affiliation:

^1^ Universidad Nacional Autónoma de México, Instituto de Ciencias del Mar y Limnología, Unidad Académica Mazatlán. P.O. Box 811, C.P. 82000, Mazatlán, Sinaloa, Mexico. ORCID: 0000-0002-1579-817X

^2^ Miembro de El Colegio de Sinaloa. Antonio Rosales 435 Pte. Culiacán, Sinaloa, Mexico

^3^ Instituto Sinaloense de Acuacultura y Pesca, Secretaría de Agricultura, Ganadería y Pesca, Gobierno del Estado de Sinaloa, Fray Servando Teresa de Mier 1870, C.P. 80129, Culiacán, Sinaloa, Mexico

^4^ Universidad Politécnica de Sinaloa, Carretera Municipal Libre Mazatlán Higueras Km 3, C.P. 82199, Mazatlán, Sinaloa, Mexico

^5^ Posgrado en Ciencias del Mar y Limnología, Universidad Nacional Autónoma de México. P.O. Box 811, C.P. 82000, Mazatlán Sinaloa, México

E-mail address of the corresponding author:

paezos@ola.icmyl.unam.mx

**Table 1SM.** Recoveries and mean concentration (± standard deviation) of metals in Reference Material DORM-4 (fish protein) and the found (mg kg^-1^ on dry weight).

| Metal | Concentration in  DORM-4* | Concentration  found | Recovery (% ) |
| --- | --- | --- | --- |
| Zn | 51.6±2.8 | 53.1±2.3 | 103.0±4.5 |
| Cu | 15.7±0.5 | 13.4±0.4 | 85.3±2.5 |
| Cd | 0.299±0.018 | 0.30±0.03 | 101.7+10.4 |
| Pb | 0.404±0.062 | 0.38±0.02 | 93.1±4.6 |

*National Research Council Canada; Dorm-4 Fish protein certified reference material for trace metals.

**Table 2SM.** Mean values (µg g^-1^ ww) of heavy metals by tissue and season determined in each dam.

| **Dam** | **Tissue** |  | **Cd** | | |  | **Cu** | | |  | **Pb** | | |  | **Zn** | | |
| --- | --- | --- | --- | --- | --- | --- | --- | --- | --- | --- | --- | --- | --- | --- | --- | --- | --- |
|  |  |  | Dry | Rain | Two seasons |  | Dry | Rain | Two seasons |  | Dry | Rain | Two seasons |  | Dry | Rain | Two seasons |
| 1 | Muscle  Liver  Gills  Guts |  | 0.005±0.002  6.37±8.04  0.026±0.010  0.85±0.83 | 0.007±0.004  5.39±4.61  0.027±0.013  0.62±0.25 | 0.006±0.003  5.88±6.46  0.025±0.011  0.74±0.62 |  | 2.1±0.4  811±800  2.3±0.7  32.5±45.2 | 0.3±0.1  1398±989  3.5±6.5  10.3±7.8 | 1.2±1.0  1104±933  2.9±4.6  21.4±33.8 |  | 0.05±0.02  0.48±0.33  0.43±0.18  6.04±4.11 | 0.02±0.01  0.35±0.27  0.21±0.09  0.29±0.12 | 0.04±0.02  0.42±0.31  0.32±0.16  3.17±4.09 |  | 5.1±1.3  43.7±28.7  22.4±9.6  19.0±6.8 | 6.8±1.4  98.8±113.4  31.6±12.7  15.5±4.0 | 5.9±1.6  71.2±86.0  27.0±12.0  17.3±5.7 |
| 2 | Muscle  Liver  Gills  Guts |  | 0.005±0.002  3.98±2.31  0.028±0.014  0.58±0.29 | 0.005±0.001  2.87±2.92  0.030±0.006  0.59±0.41 | 0.005±0.002  3.43±2.65  0.029±0.011  0.59±0.35 |  | 1.7±0.4  1097±667  2.7±0.9  18.4±11.4 | 0.3±0.1  889±893  1.6±0.6  8.0±8.3 | 1.0±0.8  993±781  2.2±0.9  13.2±11.1 |  | 0.07±0.01  0.86±0.56  0.47±0.13  6.86±2.65 | 0.02±0.01  0.34±0.18  0.11±0.06  0.37±0.45 | 0.04±0.03  0.60±0.49  0.29±0.21  3.62±3.79 |  | 6.0±1.31.0  73.4±33.5  32.2±15.6  33.6±11.9 | 6.7±1.3  89.6±37.1  32.0±13.8  24.3±7.7 | 6.3±1.2  81.5±35.7  32.1±14.5  28.9±10.9 |
| 3 | Muscle  Liver  Gills  Guts |  | 0.005±0.002  0.41±0.20  0.012±0.008  0.17±0.09 | 0.004±0.002  0.44±0.45  0.010±0.005  0.12±0.08 | 0.004±0.002  0.42±0.34  0.011±0.007  0.15±0.08 |  | 0.78±0.19  459±257  1.63±0.52  6.45±5.64 | 0.27±0.12  240±362  1.13±0.49  16.18±19.67 | 0.53±0.30  349±328  1.38±0.56  11.3±15.1 |  | 0.05±0.01  0.27±0.17  0.23±0.21  2.21±1.17 | 0.01±0.01  0.27±0.22  0.07±0.03  0.09±0.06 | 0.03±0.02  0.27±0.19  0.15±0.17  1.15±1.35 |  | 6.4±1.8  60.8±21.4  33.7±15.6  16.8±7.8 | 5.7±1.0  32.6±30.2  25.4±11.4  14.3±7.1 | 6.1±1.5  46.7±29.4  29.6±14.0  15.5±7.4 |
| 4 | Muscle  Liver  Gills  Guts |  | 0.005±0.002  2.69±1.04  0.020±0.011  0.22±0.07 | 0.004±0.001  1.05±0.83  0.024±0.013  0.24±0.20 | 0.004±0.001  1.87±1.25  0.022±0.012  0.23±0.15 |  | 2.4±1.4  704±370  2.8±1.1  15.9±14.6 | 0.2±0.1  708±730  1.8±0.5  19.2±32.8 | 1.3±1.5  706±578  2.3±1.0  17.5±25.0 |  | 0.06±0.02  0.30±0.15  3.16±4.28  1.37±0.89 | 0.01±0.01  0.11±0.10  0.14±0.05  0.12±0.04 | 0.04±0.03  0.20±0.16  1.65±3.34  0.74±0.89 |  | 5.9±0.7  57.2±23.2  24.4±10.4  20.6±7.9 | 6.0±1.3  57.7±32.8  25.6±5.4  18.0±6.9 | 5.9±1.0  57.3±27.9  25.0±8.1  19.3±20.4 |
| 5 | Muscle  Liver  Gills  Guts |  | 0.007±0.003  1.32±0.76  0.025±0.013  0.30±0.13 | 0.004±0.002  2.17±1.24  0.014±0.002  0.22±0.12 | 0.006±0.003  1.75±1.10  0.020±0.020  0.26±0.13 |  | 4.5±0.6  491±342  1.4±0.8  13.8±5.0 | 0.3±0.2  564±448  2.7±4.0  5.5±5.0 | 2.4±2.2  527±394  2.0±2.9  9.6±6.4 |  | 0.06±0.03  0.37±0.29  0.49±0.24  6.50±1.60 | 0.02±0.01  0.05±0.05  0.07±0.05  0.03±0.04 | 0.04±0.03  0.21±0.26  0.28±0.27  3.27±3.47 |  | 6.0±1.1  54.0±19.4  22.4±3.2  32.9±9.4 | 6.9±1.2  59.7±16.0  30.9±11.4  12.6±5.5 | 6.4±1.3  56.9±17.7  26.7±9.3  22.8±12.8 |
| 6 | Muscle  Liver  Gills  Guts |  | 0.004±0.002  2.78±1.17  0.018±0.003  0.69±0.43 | 0.003±0.002  0.75±0.77  0.014±0.006  0.35±0.15 | 0.004±0.002  1.46±1.34  0.015±0.006  0.47±0.32 |  | 7.3±6.6  42.7±13.2  1.7±1.6  3.8±0.9 | 0.2±0.1  149±122  0.7±0.3  8.1±6.3 | 2.7±5.1  112±110  1.1±1.1  6.6±5.4 |  | 0.02±0.01  0.25±0.07  0.30±0.08  2.24±0.53 | 0.06±0.07  0.15±0.13  0.22±0.11  1.12±0.97 | 0.04±0.06  0.18±0.12  0.25±0.11  1.51±1.00 |  | 3.9±0.4  39.1±6.6  15.0±1.1  16.2±3.3 | 5.1±0.9  22.4±13.8  23.3±7.3  20.8±6.6 | 4.7±0.9  28.2±14.2  20.4±7.1  19.2±6.0 |
| 7 | Muscle  Liver  Gills  Guts |  | 0.002±0.001  0.70±0.70  0.010±0.005  0.18±0.05 | 0.002±0.001  1.13±1.39  0.005±0.003  0.10±0.07 | 0.002±0.001  0.93±1.13  0.007±0.005  0.14±0.07 |  | 5.0±5.2  268±157  1.4±0.4  8.3±3.4 | 0.1±0.0  258±274  0.9±0.2  7.5±8.5 | 2.4±4.3  263±223  1.1±0.4  7.9±6.5 |  | 0.04±0.03  0.06±0.03  0.30±0.15  1.06±0.37 | 0.02±0.01  0.04±0.04  0.10±0.04  0.09±0.06 | 0.03±0.02  0.05±0.04  0.19±0.15  0.54±0.56 |  | 4.7±0.9  20.6±6.6  24.8±10.7  13.1±1.3 | 5.0±0.6  30.4±19.7  37.0±31.1  9.9±2.8 | 4.9±0.8  25.8±15.7  31.3±24.3  11.4±2.7 |
| 8 | Muscle  Liver  Gills  Guts |  | 0.003±0.001  1.29±1.01  0.010±0.005  0.75±0.16 | 0.002±0.001  1.39±1.14  0.009±0.004  0.45±0.36 | 0.003±0.001  1.35±1.06  0.010±0.005  0.59±0.32 |  | 1.7±1.3  277±202  1.3±1.0  7.1±3.3 | 0.1±0.1  327±229  1.0±0.4  14.0±11.9 | 0.9±1.2  304±214  1.1±0.8  10.7±9.5 |  | 0.03±0.02  0.26±0.14  0.73±0.24  11.90±3.67 | 0.05±0.04  0.26±0.24  0.43±0.26  0.42±0.32 | 0.04±0.03  0.26±0.19  0.57±0.29  5.75±6.33 |  | 5.7±1.9  24.6±9.9  25.8±9.9  26.8±7.0 | 5.8±1.0  40.6±24.3  40.7±31.3  14.9±9.8 | 5.8±1.4  33.1±20.4  33.8±24.7  20.4±10.4 |
| 9 | Muscle  Liver  Gills  Guts |  | 0.006±0.002  4.14±4.96  0.021±0.007  0.83±0.49 | 0.003±0.001  1.87±1.89  0.012±0.006  0.43±0.18 | 0.004±0.002  3.01±3.86  0.017±0.008  0.63±0.41 |  | 4.2±5.0  640±598  1.4±1.4  5.9±6.3 | 0.1±0.0  359±262  0.9±0.3  3.8±3.7 | 2.2±4.1  449±476  1.2±1.0  4.9±5.2 |  | 0.04±0.02  0.34±0.26  0.67±0.29  1.95±1.54 | 0.03±0.02  0.07±0.06  0.23±0.12  0.83±2.31 | 0.04±0.02  0.20±0.23  0.45±0.31  1.39±2.01 |  | 6.4±1.5  23.9±14.8  25.4±26.9  12.8±4.2 | 5.4±0.9  26.2±15.7  36.8±40.1  12.8±6.2 | 5.9±1.3  25.0±15.1  31.1±30.8  12.8±5.2 |
| 10 | Muscle  Liver  Gills  Guts |  | 0.005±0.001  0.67±0.41  0.014±0.006  0.14±0.05 | 0.009±0.001  0.93±0.62  0.023±0.007  0.31±0.10 | 0.007±0.003  0.80±0.53  0.019±0.008  0.23±0.12 |  | 3.7±0.5  501±243  2.2±0.4  7.5±4.6 | 0.4±0.3  268±200  1.9±0.5  6.9±4.2 | 2.0±1.7  384±249  2.1±0.5  7.2±4.3 |  | 0.06±0.04  0.36±0.16  0.33±0.13  1.30±0.57 | 0.06±0.02  0.10±0.06  0.15±0.06  0.05±0.57 | 0.06±0.03  0.23±0.18  0.24±0.14  0.68±0.75 |  | 5.3±0.8  53.6±17.8  28.2±15.2  13.1±3.9 | 6.1±1.4  40.3±21.5  36.8±12.8  15.6±3.3 | 5.6±1.2  46.9±20.5  32.5±14.5  14.3±3.8 |
| 11 | Muscle  Liver  Gills  Guts |  | 0.004±0.001  0.88±0.61  0.010±0.003  0.29±0.13 | 0.001±0.000  0.79±0.49  0.010±0.05  0.26±0.11 | 0.003±0.002  0.84±0.55  0.010±0.004  0.28±0.12 |  | 1.2±0.9  182±111  2.3±2.4  5.3±3.2 | 0.2±0.1  228±181  2.0±0.8  4.4±2.7 | 0.7±0.8  202±145  2.2±1.9  4.9±3.0 |  | 0.03±0.01  0.07±0.05  0.22±0.08  0.92±0.58 | 0.02±0.00  0.04±0.03  0.13±0.04  0.38±0.20 | 0.02±0.01  0.06±0.05  0.18±0.08  0.68±0.52 |  | 3.9±0.6  16.9±6.1  18.8±5.8  12.7±2.5 | 4.2±0.6  18.0±9.1  40.7±31.7  12.5±1.4 | 4.0±0.6  17.4±7.4  28.5±23.8  12.6±2.1 |

**Table 3SM1**. Morphometric measurements (mean±SE) of dam samples of tilapia by season and sex.

|  |  |  |  | Weight (g) | | |  | Total Length (cm) | | |
| --- | --- | --- | --- | --- | --- | --- | --- | --- | --- | --- |
| Dam | Season | n |  | Female (n) | Male (n) | Total |  | Female (n) | Male (n) | Total |
| 1 | Rain Dry | 15  15 |  | 414.5±24.7 (4)  501.3±31.0 (8) | 497.3±38.8 (11) 524.6±14.2 (7) | 475.2±30.3  512.1±17.5 |  | 27.0±0.8 (4)  30.4±0.7 (8) | 28.9±0.8 (11)  31.3±0.6 (7) | 28.4±0.6  30.8±0.5 |
| 2 | Rain Dry | 15  15 |  | 124.5±9.0 (4)  196.0±10.7 (4) | 140.0±11.9 (11) 206.5±12.5 (11) | 135.9±9.1  203.7±9.5 |  | 19.0±0.4 (4)  21.8±0.9 (4) | 19.8±0.5 (11)  22.9±0.4 (11) | 19.6±0.4  22.6±0.4 |
| 3 | Rain Dry | 15  15 |  | 322.0 (1)  183.5±4.6 (4) | 287.6±25.3 (14) 230.7±8.7 (11) | 289.9±23.6  218.1±8.5 |  | 25.0 (1)  21.3±0.5 (4) | 24.6±0.4 (14)  23.5±0.3 (11) | 24.7±0.4  22.9±0.4 |
| 4 | Rain Dry | 15  15 |  | 292.0±10.9 (11)  310.0±35.0 (8) | 366.0±58.9 (4) 349.4±27.7 (7) | 311.7±18.4  328.4.5±22.5 |  | 24.9±0.4 (11)  25.0±0.9 (8) | 27.3±2.1 (4)  27.1±1.1 (7) | 25.5±0.6  26.0±0.7 |
| 5 | Rain Dry | 15  15 |  | 304.0±18.0 (2)  308.3±9.3 (7) | 318.9±9.4 (13) 302.8±6.7 (8) | 316.9±8.4  305.2±6.5 |  | 25.5±0.5 (2)  24.9±0.5 (7) | 25.8±0.3 (13)  24.8±0.3 (8) | 25.7±0.2  24.8±0.3 |
| 6 | Rain  Dry | 15  12 |  | 160.5±9.2 (4)  126.3±2.1 (6) | 163.3±7.7 (11) 126.3±2.1 (6) | 162.5±6.0  126.3±1.3 |  | 21.0±0.6 (4)  19.0±0.0 (6) | 21.0±0.4 (11)  19.0±0.0 (6) | 21.0±0.3  19.0±0.0 |
| 7 | Rain  Dry | 15  13 |  | 260.5±19.0 (4)  358.0±41.4 (3) | 250.4±16.0 (11) 389.8±26.7 (10) | 253.1±12.5  382.5±22.2 |  | 23.5±0.6 (4)  27.3±0.9 (3) | 23.9±0.5 (11)  27.1±0.6 (10) | 23.8±0.4  27.2±0.5 |
| 8 | Rain  Dry | 15  13 |  | 153.0±10.5 (3)  244.5±9.6 (4) | 162.5±12.8 (12) 245.9±12.0 (9) | 160.6±10.3  245.5±8.6 |  | 21.0±1.0 (3)  23.3±0.3 (4) | 21.4±0.8 (12)  24.0±0.6 (9) | 21.3±0.6  23.8±0.4 |
| 9 | Rain Dry | 15  15 |  | 332.3±23.1 (3)  356.8±23.3 (10) | 353.3±21.5 (12) 434.0±30.2 (5) | 349.1±17.6  382.5±20.3 |  | 25.0±0.6 (3)  26.2±0.3 (10) | 26.1±0.6 (12)  28.4±1.3 (5) | 25.9±0.5  26.9±0.5 |
| 10 | Rainy Dry | 15  15 |  | 459.7±33.7 (7)  496.9±19.4 (7) | 495.3±21.6 (8) 492.5±21.0 (8) | 478.7±19.3  494.5±13.9 |  | 27.9±0.7 (7)  27.9±0.6 (7) | 28.4±0.5 (8)  28.8±0.6 (8) | 28.1±0.4  28.3±0.4 |
| 11 | Rain  Dry | 12  15 |  | 720.7±28.8 (6)  723.6±56.5 (7) | 856.0±49.6 (6) 940.5±55.0 (8) | 788.3±34.1  839.3±47.8 |  | 32.9±0.2 (6)  32.9±0.9 (7) | 35.3±0.6 (6)  36.9±0.7 (8) | 34.1±0.5  35.0±0.7 |

The location and name of each dam is showed in Fig. 1 and Table 1. SE= standard error; n= sampled organisms; ( ) = number of organisms of each sex.

**Tables 3M2-3SM12 and figures 3SM1-3SM23.** Spearman correlations (r) among metal concentrations and body size of tilapia in dams

**Dam 1:**

The only significant correlations were found between total length (TL) and Cu in muscle and guts (p<0.05; Fig 3S1), as well as Pb in muscle, gills and guts (p<0.05; Fig. 3S2). Similar patterns of associations were found between weight and Cu in muscle (r=0.49; p<0.01), Cu in guts (r=0.48; p<0.01, not showed); for Pb in gills, a significant correlation was found with weight (r=0.38; p<0.05, not showed).

**Fig. 3SM1**. Correlations between TL (cm) and Cu (µg g^-1^) in muscle and guts (○ males and ● females) in dam 1.

**Fig. 3SM2.** Correlations between TL and Pb (µg g^-1^) in muscle, guts and gills (○ males and ● females) in dam 1.

**Table 3SM2**. Spearman correlations (r) among metal concentrations in muscle, liver, gills and guts of tilapia at dam 1: significant (p<0.05), and NS=not significant (p>0.05) values.

| Metal  tissu | Cd m | Cd  gill | Cd gu | Cd liv | Cu m | Cu gill | Cu gu | Cu liv | Pb mu | Pb gill | Pb gu | Pb liv | Zn mu | Zn gill | Zn gu | Zn liv |
| --- | --- | --- | --- | --- | --- | --- | --- | --- | --- | --- | --- | --- | --- | --- | --- | --- |
| Cd muscle | - | NS | NS | NS | NS | NS | NS | NS | NS | NS | -0.48 | NS | NS | NS | NS | NS |
| Cd gills | NS | - | 0.41 | 0.45 | NS | 0.46 | NS | 0.38 | NS | NS | NS | NS | NS | NS | NS | NS |
| Cd guts | NS | 0.41 | - | 0.48 | NS | 0.37 | NS | 0.42 | NS | NS | NS | NS | NS | NS | NS | 0.40 |
| Cd liver | NS | 0.45 | 0.48 | - | NS | 0.40 | NS | 0.87 | NS | NS | NS | NS | NS | 0.40 | NS | 0.66 |
| Cu muscle | NS | NS | NS | NS | - | 0.42 | 0.44 | NS | 0.71 | 0.76 | 0.68 | NS | NS | -0.38 | NS | -0.37 |
| Cu gills | NS | 0.46 | 0.37 | 0.40 | 0.42 | - | NS | NS | NS | 0.57 | NS | 0.38 | NS | NS | NS | NS |
| Cu guts | NS | NS | NS | NS | 0.44 | NS | - | NS | NS | NS | 0.38 | NS | NS | NS | NS | NS |
| Cu liver | NS | 0.38 | 0.42 | 0.87 | NS | NS | NS | - | NS | NS | NS | NS | 0.37 | 0.58 | NS | 0.60 |
| Pb muscle | NS | NS | NS | NS | 0.71 | NS | NS | NS | - | 0.65 | 0.56 | NS | NS | NS | NS | NS |
| Pb gills | NS | NS | NS | NS | 0.76 | 0.57 | NS | NS | 0.65 | - | 0.56 | NS | NS | NS | NS | NS |
| Pb guts | 0.48 | NS | NS | NS | 0.68 | NS | 0.38 | NS | 0.56 | 0.63 | - | 0.39 | -0.54 | -0.37 | 0.55 | -0.44 |
| Pb liver | NS | NS | NS | NS | NS | 0.38 | NS | NS | NS | NS | 0.39 | - | NS | NS | NS | 0.45 |
| Zn muscle | NS | NS | NS | NS | NS | NS | NS | 0.37 | NS | NS | -0.54 | NS | - | 0.49 | NS | NS |
| Zn gills | 0.40 | NS | NS | 0.40 | -0.38 | NS | NS | 0.58 | NS | NS | -0.37 | NS | 0.49 | - | NS | NS |
| Zn guts | NS | NS | NS | NS | NS | NS | NS | NS | NS | NS | 0.55 | NS | NS | NS | - | NS |
| Zn liver | NS | NS | 0.40 | 0.66 | -0.37 | NS | NS | 0.60 | NS | NS | -0.44 | 0.45 | NS | NS | NS | - |

**Dam 2:**

The only significant (p<0.05) correlations were found between TL and Cd in muscle and gills (Fig. 3S3), Cu in muscle (Fig. 3S4), as well as Pb in all studied tissues (Fig. 3S5) and Zn in liver (Fig 3S6). Similar patterns of association were found between weight and Cu in muscle (r=0.60; p<0.001) and Pb in muscle (r=0.60; p<0.001), Pb in gills (r=0.55; p<0.01), Pb in guts (r=0.59; p<0.001) and Pb in liver (r= 0.40; p<0.05), as well as Zn in liver (r= -0.54; p<0.05).

**Fig. 3SM3**. Negative associations among TL (cm) and Cd (µg g^-1^) in muscle and gills (○ males and ● females) in dam 2.

**Fig. 3SM4**. Correlations between TL and Cu (µg g^-1^) in muscle (○ males and ● females) in dam 2.

**Fig. 3SM5**. Correlations between TL and Pb (µg g^-1^) in muscle, gills, guts and liver (○ males and ● females) in dam 2.

**Fig. 3SM6**. Negative correlation between TL and Zn in liver (µg g^-1^) (○ males and ● females) in dam 2.

**Table 3SM3**. Spearman correlations (r) among elements concentrations in muscle, liver, gills and guts of tilapia at dam 2: significant (p<0.05), and NS = not significant (p>0.05) values.

| Metal  tissue | Cd muscle | Cd  gills | Cd guts | Cd liver | Cu muscle | Cu gills | Cu guts | Cu liver | Pb muscle | Pb gills | Pb guts | Pb liver | Zn muscle | Zn gills | Zn guts | Zn liver |
| --- | --- | --- | --- | --- | --- | --- | --- | --- | --- | --- | --- | --- | --- | --- | --- | --- |
| Cd muscle | - | 0.53 | NS | NS | NS | NS | NS | NS | NS | NS | NS | NS | NS | NS | NS | NS |
| Cd gills | 0.53 | - | 0.41 | NS | NS | NS | NS | NS | -0.40 | NS | NS | NS | NS | 0.47 | NS | NS |
| Cd guts | NS | NS | - | 0.49 | NS | NS | 0.37 | 0.43 | NS | NS | NS | NS | NS | NS | 0.44 | NS |
| Cd liver | NS | NS | 0.49 | - | NS | 0.61 | 0.40 | 0.69 | 0.36 | 0.56 | 0.40 | 0.61 | NS | NS | NS | 0.58 |
| Cu muscle | NS | NS | NS | NS | - | 0.59 | 0.60 | NS | 0.84 | 0.79 | 0.84 | 0.61 | NS | NS | 0.45 | NS |
| Cu gills | NS | NS | NS | 0.61 | 0.59 | - | 0.52 | 0.36 | 0.53 | 0.78 | 0.65 | 0.62 | NS | 0.57 | 0.49 | 0.40 |
| Cu guts | NS | NS | 0.37 | 0.40 | 0.60 | 0.52 | - | NS | 0.64 | 0.54 | 0.69 | 0.50 | NS | NS | 0.51 | NS |
| Cu liver | NS | NS | 0.43 | 0.69 | NS | 0.36 | NS | - | NS | NS | NS | NS | NS | NS | NS | NS |
| Pb muscle | NS | -0.40 | NS | 0.36 | 0.84 | 0.63 | 0.64 | NS | - | 0.78 | 0.79 | 0.60 | NS | NS | 0.53 | NS |
| Pb gills | NS | NS | NS | 0.56 | 0.79 | 0.78 | 0.54 | NS | 0.78 | - | 0.78 | 0.70 | NS | NS | 0.50 | NS |
| Pb guts | NS | NS | NS | 0.40 | 0.84 | 0.66 | 0.69 | NS | 0.79 | 0.78 | - | 0.67 | NS | NS | 0.59 | NS |
| Pb liver | NS | NS | NS | 0.61 | 0.61 | 0.62 | 0.50 | NS | 0.59 | 0.70 | 0.67 | - | NS | NS | 0.47 | NS |
| Zn muscle | NS | NS | NS | NS | NS | NS | NS | NS | NS | NS | NS | NS | - | NS | NS | NS |
| Zn gills | NS | 0.47 | NS | NS | NS | 0.57 | NS | NS | NS | NS | NS | NS | NS | - | NS | NS |
| Zn guts | NS | NS | 0.44 | NS | 0.45 | 0.48 | 0.51 | NS | 0.53 | 0.50 | 0.59 | 0.47 | NS | NS | - | NS |
| Zn liver | NS | NS | NS | 0.58 | NS | 0.40 | NS | NS | NS | NS | NS | NS | NS | NS | NS | - |

**Dam 3:**

Significant negative correlations (p<0.05) were found between total length (TL) and Cu in muscle, Pb in muscle, Pb in gills and Zn in muscle (Fig. 3S7). Similar patterns of associations were found between weight and Cu in muscle (r= -0.70; p<0.0001), Cu in liver (r= -0.46; p<0.05), Pb in muscle (r= -0.68; p<0.0001), Pb in gills (r= -0.50; p<0.01), Zn in muscle (r= -0.46; p<0.01) and Zn in liver (r= -0.50; p<0.01).

**Fig. 3SM7**. Negative associations between TL (cm) and Cu (µg g^-1^) in muscle, Pb (µg g^-1^) in muscle and gills and Zn in muscle (µg g^-1^) (○ males and ● females) in dam 3.

**Table 3SM4**. Spearman correlations (r) among elements concentrations in muscle, liver, gills and guts of tilapia at dam 3: significant (p<0.05) and NS = not significant (p>0.05) values.

| Metal  tissue | Cd muscle | Cd  gills | Cd guts | Cd liver | Cu muscle | Cu gills | Cu guts | Cu liver | Pb muscle | Pb gills | Pb guts | Pb liver | Zn muscle | Zn gills | Zn guts | Zn liver |
| --- | --- | --- | --- | --- | --- | --- | --- | --- | --- | --- | --- | --- | --- | --- | --- | --- |
| Cd muscle | - | NS | NS | NS | NS | NS | -0.44 | NS | 0.48 | NS | NS | NS | 0.64 | NS | NS | NS |
| Cd gills | NS | - | NS | NS | NS | 0.54 | NS | NS | NS | 0.42 | NS | NS | NS | 0.60 | NS | NS |
| Cd guts | NS | NS | - | NS | NS | NS | 0.40 | NS | 0.38 | NS | 0.58 | NS | NS | NS | 0.74 | NS |
| Cd liver | NS | NS | NS | - | NS | NS | NS | 0.71 | NS | NS | NS | 0.66 | NS | NS | NS | 0.77 |
| Cu muscle | NS | NS | NS | NS | - | 0.41 | NS | 0.40 | 0.87 | 0.70 | 0.67 | NS | 0.60 | NS | NS | 0.49 |
| Cu gills | NS | 0.54 | NS | NS | 0.41 | - | NS | NS | NS | 0.70 | 0.38 | NS | NS | 0.69 | NS | NS |
| Cu guts | -0.44 | NS | 0.40 | NS | NS | NS | - | NS | NS | NS | NS | NS | NS | NS | 0.42 | NS |
| Cu liver | NS | NS | NS | 0.71 | 0.40 | NS | NS | - | 0.46 | NS | NS | 0.49 | NS | NS | NS | 0.81 |
| Pb muscle | 0.48 | NS | 0.38 | NS | 0.87 | NS | NS | 0.46 | - | 0.73 | 0.73 | NS | 0.60 | NS | NS | 0.65 |
| Pb gills | NS | 0.43 | NS | NS | 0.790 | 0.70 | NS | NS | 0.73 | - | 0.68 | NS | 0.38 | 0.47 | NS | NS |
| Pb guts | NS | NS | 0.58 | NS | 0.67 | 0.38 | NS | NS | 0.73 | 0.68 | - | NS | NS | NS | 0.40 | 0.41 |
| Pb liver | NS | NS | NS | 0.66 | NS | NS | NS | 0.49 | NS | NS | NS | - | NS | NS | NS | 0.67 |
| Zn muscle | 0.64 | NS | NS | NS | 0.60 | NS | NS | NS | 0.60 | 0.38 | NS | NS | - | NS | NS | NS |
| Zn gills | NS | 0.60 | NS | NS | NS | 0.69 | NS | NS | NS | 0.47 | NS | NS | NS | - | NS | NS |
| Zn guts | NS | NS | 0.74 | NS | NS | NS | 0.42 | NS | NS | NS | 0.40 | NS | NS | NS | - | NS |
| Zn liver | NS | NS | NS | 0.77 | 0.49 | NS | NS | 0.81 | 0.55 | NS | 0.41 | 0.67 | NS | NS | NS | - |

**Dam 4:**

The only negative significant correlation (p<0.05) was found between total length (TL) and Cd in gills (Fig. 3S8), and positive associations were found between TL and Cd in liver and Cu in guts (Fig. 3S9). Patterns of association were found between weight and Cd in gills (r= -0.42; p<0.05) and liver (r= 0.40; p<0.05) as well as Cu in guts (r= 0.45; p<0.05) and in liver (r= 0.38; p<0.05). Levels of Pb in liver (r= 0.40; p<0.05) and Zn in guts (r= 0.41; p<0.05) presented significant positive correlations with weight.

**Fig. 3SM8**. Negative association among TL (cm) and Cd (µg g^-1^) in gills (○ males and ● females) in dam 4.

**Fig. 3SM9**. Positive correlations between TL and Cd (µg g^-1^) in liver and Cu in guts (µg g^-1^) (○ males and ● females) in dam 4.

**Table 3SM5**. Spearman correlations (r) among elements concentrations in muscle, liver, gills and guts of tilapia at dam 4: significant (p<0.05) and NS = not significant (p>0.05) values.

| Element/  tissue | Cd muscle | Cd  gills | Cd guts | Cd liver | Cu muscle | Cu gills | Cu guts | Cu liver | Pb muscle | Pb gills | Pb guts | Pb liver | Zn muscle | Zn gills | Zn guts | Zn liver |
| --- | --- | --- | --- | --- | --- | --- | --- | --- | --- | --- | --- | --- | --- | --- | --- | --- |
| Cd muscle | - | NS | NS | 0.49 | 0.41 | 0.46 | NS | NS | 0.60 | 0.47 | 0.46 | NS | NS | NS | NS | NS |
| Cd gills | NS | - | NS | NS | NS | NS | NS | NS | NS | NS | NS | NS | NS | 0.61 | NS | NS |
| Cd guts | NS | NS | - | NS | NS | NS | 0.71 | NS | NS | NS | NS | NS | NS | NS | 0.60 | NS |
| Cd liver | 0.49 | NS | NS | - | 0.67 | NS | NS | 0.61 | 0.51 | 0.55 | 0.40 | 0.82 | NS | NS | NS | 0.55 |
| Cu muscle | 0.41 | NS | NS | 0.67 | - | 0.45 | NS | NS | 0.73 | 0.69 | 0.73 | 0.71 | NS | NS | NS | NS |
| Cu gills | 0.46 | NS | NS | NS | 0.45 | - | NS | NS | 0.38 | 0.59 | 0.44 | NS | NS | 0.47 | NS | NS |
| Cu guts | NS | NS | 0.71 | NS | NS | NS | - | NS | NS | NS | NS | NS | NS | NS | 0.59 | NS |
| Cu liver | NS | NS | NS | 0.61 | NS | NS | NS | - | NS | NS | NS | 0.53 | 0.44 | NS | NS | 0.74 |
| Pb muscle | 0.60 | NS | NS | 0.51 | 0.73 | 0.38 | NS | NS | - | 0.76 | 0.80 | 0.48 | NS | NS | NS | NS |
| Pb gills | 0.47 | NS | NS | 0.55 | 0.69 | 0.59 | NS | NS | 0.76 | - | 0.70 | 0.50 | NS | NS | NS | NS |
| Pb guts | 0.46 | NS | NS | 0.40 | 0.73 | 0.44 | NS | NS | 0.80 | 0.70 | - | 0.45 | NS | NS | NS | NS |
| Pb liver | NS | NS | NS | 0.82 | 0.71 | NS | NS | 0.53 | 0.48 | 0.51 | 0.45 | - | NS | NS | NS | 0.42 |
| Zn muscle | NS | NS | NS | NS | NS | NS | NS | 0.44 | NS | NS | NS | NS | - | NS | NS | 0.43 |
| Zn gills | NS | 0.61 | NS | NS | NS | 0.47 | NS | NS | NS | NS | NS | NS | NS | - | NS | NS |
| Zn guts | NS | NS | 0.60 | NS | NS | NS | 0.59 | NS | NS | NS | NS | NS | NS | NS | - | NS |
| Zn liver | NS | NS | NS | 0.55 | NS | NS | NS | 0.74 | NS | NS | NS | 0.42 | 0.43 | NS | NS | - |

**Dam 5:**

The only significant correlations were found between total length (TL) and Cu in muscle (Fig 3S10), as well as Pb in all studied tissues (Fig. 3S11). Weight only was negative associated with Pb in gills (r= -0.36; p<0.05).

**Fig. 3SM10**. Associations among TL (cm) and Cu (µg g^-1^) in muscle and guts (○ males and ● females) in dam 5.

**Fig. 3SM11**. Correlations between TL and Pb (µg g^-1^) in muscle, liver, guts and gills (○ males and ● females) in dam 5.

**Table 3SM6**. Spearman correlations (r) among elements concentrations in muscle, liver, gills and guts of tilapia at dam 5: significant (p<0.05) and NS = not significant (p>0.05) values.

| Metal  tissue | Cd muscle | Cd  gills | Cd guts | Cd liver | Cu muscle | Cu gills | Cu guts | Cu liver | Pb muscle | Pb gills | Pb guts | Pb liver | Zn muscle | Zn gills | Zn guts | Zn liver |
| --- | --- | --- | --- | --- | --- | --- | --- | --- | --- | --- | --- | --- | --- | --- | --- | --- |
| Cd muscle | - | NS | 0.47 | NS | 0.79 | NS | 0.52 | NS | 0.72 | 0.55 | 0.64 | 0.62 | NS | NS | 0.62 | NS |
| Cd gills | NS | - | NS | NS | NS | NS | NS | NS | NS | 0.42 | NS | NS | NS | NS | NS | NS |
| Cd guts | 0.47 | NS | - | NS | 0.41 | NS | 0.43 | NS | NS | NS | 0.57 | NS | NS | NS | 0.39 | NS |
| Cd liver | NS | NS | NS | - | NS | 0.61 | NS | 0.71 | NS | NS | NS | NS | NS | 0.64 | -0.37 | 0.72 |
| Cu muscle | 0.79 | NS | 0.41 | NS | - | NS | 0.64 | NS | 0.88 | 0.77 | 0.81 | 0.73 | NS | -0.44 | 0.73 | NS |
| Cu gills | NS | NS | NS | 0.61 | NS | - | NS | 0.41 | NS | NS | NS | NS | 0.48 | 0.75 | -0.43 | NS |
| Cu guts | 0.52 | NS | 0.43 | NS | 0.64 | NS | - | NS | 0.57 | 0.61 | 0.82 | 0.50 | NS | NS | 0.81 | NS |
| Cu liver | NS | NS | NS | 0.71 | NS | 0.41 | NS | - | NS | NS | NS | NS | NS | 0.43 | NS | 0.63 |
| Pb muscle | 0.72 | NS | NS | NS | 0.88 | NS | 0.57 | NS | - | 0.78 | 0.73 | 0.78 | NS | -0.37 | 0.75 | NS |
| Pb gills | 0.55 | 0.42 | NS | NS | 0.77 | NS | 0.61 | NS | 0.78 | - | 0.77 | 0.79 | NS | NS | 0.73 | NS |
| Pb guts | 0.64 | NS | 0.67 | NS | 0.81 | NS | 0.82 | NS | 0.73 | 0.77 | - | 0.70 | NS | -0.48 | 0.90 | NS |
| Pb liver | 0.62 | NS | NS | NS | 0.73 | NS | 0.50 | NS | 0.78 | 0.79 | 0.70 | - | -0.38 | -0.40 | 0.66 | NS |
| Zn muscle | NS | NS | NS | NS | NS | 0.48 | NS | NS | NS | NS | NS | -0.38 | - | 0.38 | -0.45 | NS |
| Zn gills | NS | NS | NS | 0.64 | -0.44 | 0.75 | NS | 0.43 | -0.37 | NS | -0.48 | -0.40 | 0.30 | - | -0.40 | 0.50 |
| Zn guts | 0.62 | NS | 0.39 | -0.37 | 0.76 | -0.43 | 0.80 | NS | 0.75 | 0.73 | 0.90 | 0.66 | -0.45 | -0.48 | - | NS |
| Zn liver | NS | NS | NS | 0.72 | NS | NS | NS | 0.63 | NS | NS | NS | NS | NS | 0.50 | NS | - |

**Dam 6:**

Significant correlations were found between TL and Cd in guts and liver (Fig. 3S12), as well as TL and Cu muscle and liver (Fig. 3S13) and TL and Zn in muscle, gills and liver (Fig. 3S14). Weight followed the same correlations patterns as TL. The significant associations found were between weight and Cd in guts (r= -0.51; p<0.05) and liver (r= -0.46; p<0.05), Cu in muscle (r= -0.58; p<0.01) and in liver (r= 0.45; p<0.05), and Zn in gills (r= 0.46; p<0.05) and guts (r= -0.52; p<0.01).

**Fig. 3SM12**. Associations among TL (cm) and Cd (µg g^-1^) in guts and liver (○ males and ● females) in dam 6.

**Fig. 3SM13**. Correlations between TL and Cu (µg g^-1^) in muscle, liver and liver (○ males and ● females) in dam 6.

**Fig. 3SM14**. Correlations between TL and Zn (µg g^-1^) in muscle, gills and liver (○ males and ● females) in dam 6.

**Table 3SM7**. Spearman correlations (r) among elements concentrations in muscle, liver, gills and guts of tilapia at dam 6: significance (p<0.05) and NS = not significant (p>0.05) values.

| Metal  tissue | Cd muscle | Cd  gills | Cd guts | Cd liver | Cu muscle | Cu gills | Cu guts | Cu liver | Pb muscle | Pb gills | Pb guts | Pb liver | Zn muscle | Zn gills | Zn guts | Zn liver |
| --- | --- | --- | --- | --- | --- | --- | --- | --- | --- | --- | --- | --- | --- | --- | --- | --- |
| Cd muscle | - | NS | NS | NS | NS | NS | NS | NS | NS | NS | NS | NS | NS | NS | NS | NS |
| Cd gills | NS | - | NS | NS | NS | 0.81 | -0.42 | -0.43 | -0.48 | 0.48 | NS | NS | -0.54 | NS | -0.67 | NS |
| Cd guts | NS | NS | - | 0.66 | 0.48 | NS | NS | NS | NS | NS | NS | 0.46 | NS | NS | NS | 0.71 |
| Cd liver | NS | NS | 0.66 | - | 0.63 | NS | NS | NS | -0.53 | NS | 0.44 | 0.78 | -0.61 | -0.59 | NS | 0.84 |
| Cu muscle | NS | NS | 0.48 | 0.63 | - | NS | NS | -0.47 | NS | NS | 0.49 | NS | -0.44 | -0.71 | NS | 0.45 |
| Cu gills | NS | 0.81 | NS | NS | NS | - | NS | NS | NS | 0.67 | NS | NS | -0.43 | NS | -0.63 | NS |
| Cu guts | NS | -0.42 | NS | NS | NS | NS | - | 0.62 | NS | NS | NS | NS | 0.61 | NS | 0.62 | NS |
| Cu liver | NS | -0.43 | NS | NS | -0.47 | NS | 0.62 | - | NS | NS | NS | NS | 0.42 | NS | NS | NS |
| Pb muscle | NS | -0.48 | NS | -0.53 | NS | NS | NS | NS | - | NS | NS | -0.49 | 0.61 | NS | NS | -0.43 |
| Pb gills | NS | 0.48 | NS | NS | NS | 0.67 | NS | NS | NS | - | NS | NS | NS | NS | -0.44 | NS |
| Pb guts | NS | NS | NS | 0.44 | 0.49 | NS | NS | NS | NS | NS | - | 0.47 | -0.47 | -0.66 | NS | NS |
| Pb liver | NS | NS | 0.46 | 0.48 | NS | NS | NS | NS | -0.49 | NS | 0.47 | - | -0.43 | -0.47 | NS | 0.71 |
| Zn muscle | NS | -0.54 | NS | -0.61 | -0.44 | -0.43 | 0.61 | 0.42 | 0.61 | NS | -0.48 | -0.43 | - | 0.43 | 0.44 | -0.49 |
| Zn gills | NS | NS | NS | -0.59 | -0.71 | NS | NS | NS | NS | NS | -0.66 | -0.47 | 0.43 | - | NS | NS |
| Zn guts | NS | -0.67 | NS | NS | NS | -0.63 | 0.62 | NS | NS | -0.44 | NS | NS | 0.44 | NS | - | NS |
| Zn liver | NS | NS | 0.71 | 0.84 | 0.45 | NS | NS | NS | -0.43 | NS | NS | 0.71 | -0.49 | NS | NS | - |

**Dam 7:**

Significant correlations were found between TL and Cd (Fig. 3S15) and Pb (Fig. 3S16) in gills and guts; also TL was associated with Cu in muscle, gills and guts (Fig. 3S17) and with Zn in guts (Fig. 3S18). The same patterns of association were found between weight and Cd in gills and guts, Cu in muscle, gills and guts, Pb in gills and guts and Zn in guts, with Spearman correlations coefficients from 0.43 to 0.69 (p<0.05).

**Fig. 3SM15**. Associations among TL (cm) and Cd (µg g^-1^) in gills and guts (○ males and ● females) in dam 7.

**Fig. 3SM16**. Correlations between TL and Pb (µg g^-1^) in gills and guts (○ males and ● females) in dam 7.

**Fig. 3SM17**. Correlations between TL and Cu (µg g^-1^) in muscle, gills and guts (○ males and ● females) in dam 7.

**Fig. 3SM18**. Correlation between TL and Zn in guts (µg g^-1^) (○ males and ● females) in dam 7.

**Table 3SM8**. Spearman correlations (r) among elements concentrations in muscle, liver, gills and guts of tilapia at dam 7: significant (p<0.05) and NS = not significant (p>0.05) values.

| Metal  tissue | Cd muscle | Cd  gills | Cd guts | Cd liver | Cu muscle | Cu gills | Cu guts | Cu liver | Pb muscle | Pb gills | Pb guts | Pb liver | Zn muscle | Zn gills | Zn guts | Zn liver |
| --- | --- | --- | --- | --- | --- | --- | --- | --- | --- | --- | --- | --- | --- | --- | --- | --- |
| Cd muscle | - | NS | NS | NS | NS | NS | NS | NS | NS | NS | NS | NS | NS | NS | NS | NS |
| Cd gills | NS | - | NS | NS | 0.57 | 0.62 | NS | NS | NS | 0.57 | 0.54 | 0.38 | NS | NS | NS | NS |
| Cd guts | NS | NS | - | 0.49 | 0.51 | 0.58 | 0.70 | 0.51 | NS | 0.51 | 0.54 | NS | NS | NS | 0.62 | NS |
| Cd liver | NS | NS | 0.49 | - | NS | NS | NS | 0.79 | NS | NS | NS | 0.65 | NS | NS | NS | 0.75 |
| Cu muscle | NS | 0.57 | 0.51 | NS | - | 0.58 | 0.40 | NS | NS | 0.74 | 0.69 | 0.52 | NS | NS | 0.40 | NS |
| Cu gills | NS | 0.62 | 0.58 | NS | 0.58 | - | 0.49 | 0.39 | NS | 0.71 | 0.71 | NS | NS | NS | 0.58 | NS |
| Cu guts | NS | NS | 0.70 | NS | 0.40 | 0.49 | - | 0.45 | NS | 0.45 | NS | NS | NS | NS | NS | NS |
| Cu liver | NS | NS | 0.51 | 0.79 | NS | 0.39 | 0.45 | - | NS | NS | NS | 0.76 | NS | NS | NS | 0.80 |
| Pb muscle | NS | NS | NS | NS | NS | NS | NS | NS | - | NS | NS | NS | 0.49 | NS | NS | NS |
| Pb gills | NS | 0.57 | 0.61 | NS | 0.74 | 0.71 | 0.45 | NS | NS | - | 0.76 | NS | NS | NS | 0.51 | NS |
| Pb guts | NS | 0.54 | 0.54 | NS | 0.69 | 0.71 | NS | NS | NS | 0.76 | - | 0.46 | NS | NS | 0.77 | NS |
| Pb liver | NS | 0.38 | NS | 0.65 | 0.52 | NS | NS | 0.76 | NS | NS | 0.46 | - | NS | NS | 0.43 | 0.67 |
| Zn muscle | NS | NS | NS | NS | NS | NS | NS | NS | 0.49 | NS | NS | NS | - | NS | NS | NS |
| Zn gills | NS | NS | NS | NS | NS | NS | NS | NS | NS | NS | NS | NS | NS | - | NS | NS |
| Zn guts | NS | NS | 0.62 | NS | 0.40 | 0.58 | NS | NS | NS | 0.51 | 0.77 | 0.2 | NS | NS | - | NS |
| Zn liver | NS | NS | NS | 0.75 | NS | NS | NS | 0.80 | NS | NS | NS | 0.67 | NS | NS | NS | - |

**Dam 8:**

Three significant correlations were found between the studied variables and TL, only with Cu in muscle (Fig. 3S19) and Pb (Fig. 3S20) in muscle and guts. Regarding weight, there were found significant (p<0.05) correlations between this variable and Cd (r= 0.42), Cu (r= 0.66) and Pb (r= -0.43) in muscle and Pb (r= 0.67) and Zn (r= 0.45) in guts.

**Fig. 3SM19**. Association between TL and Cu (µg g^-1^) in muscle (○ males and ● females) in dam 8.

**Fig. 3SM20**. Correlations between TL and Pb (µg g^-1^) in muscle and guts (○ males and ● females) in dam 8.

**Table 3SM9**. Spearman correlations (r) among elements concentrations in muscle, liver, gills and guts of tilapia at dam 8: significant (p<0.05) and NS = not significant (p>0.05) values.

| Metal  tissue | Cd muscle | Cd  gills | Cd guts | Cd liver | Cu muscle | Cu gills | Cu guts | Cu liver | Pb muscle | Pb gills | Pb guts | Pb liver | Zn muscle | Zn gills | Zn guts | Zn liver |
| --- | --- | --- | --- | --- | --- | --- | --- | --- | --- | --- | --- | --- | --- | --- | --- | --- |
| Cd muscle | - | NS | NS | NS | 0.45 | NS | NS | NS | NS | 0.62 | 0.47 | NS | -0.44 | NS | NS | NS |
| Cd gills | NS | - | NS | NS | NS | 0.55 | NS | NS | NS | 0.41 | NS | NS | NS | NS | NS | NS |
| Cd guts | NS | NS | - | NS | 0.52 | NS | 0.42 | NS | NS | 0.48 | 0.69 | NS | NS | NS | 0.72 | NS |
| Cd liver | NS | NS | NS | - | NS | NS | NS | 0.76 | NS | NS | NS | 0.65 | NS | 0.41 | NS | 0.73 |
| Cu muscle | 0.45 | NS | 0.52 | NS | - | NS | NS | NS | NS | 0.49 | 0.80 | NS | NS | NS | 0.62 | -0.39 |
| Cu gills | NS | 0.55 | NS | NS | NS | - | NS | NS | NS | NS | NS | 0.42 | NS | NS | NS | 0.40 |
| Cu guts | NS | NS | 0.42 | NS | NS | NS | - | NS | NS | NS | NS | NS | NS | NS | NS | NS |
| Cu liver | NS | NS | NS | 0.76 | NS | NS | NS | - | NS | NS | NS | 0.55 | NS | 0.41 | NS | 0.64 |
| Pb muscle | NS | NS | NS | NS | NS | NS | NS | NS | - | NS | NS | NS | NS | NS | NS | NS |
| Pb gills | 0.62 | 0.41 | 0.48 | NS | 0.49 | NS | NS | NS | NS | - | 0.61 | 0.39 | NS | NS | 0.50 | NS |
| Pb guts | 0.47 | NS | 0.69 | NS | 0.80 | NS | NS | NS | NS | 0.61 | - | NS | -0.39 | NS | 0.85 | NS |
| Pb liver | NS | NS | NS | 0.65 | NS | 0.42 | NS | 0.55 | NS | 0.39 | NS | - | -0.39 | NS | NS | 0.75 |
| Zn muscle | -0.44 | NS | NS | NS | NS | NS | NS | NS | NS | NS | -0.39 | -0.39 | - | NS | NS | NS |
| Zn gills | NS | NS | NS | 0.41 | NS | NS | NS | 0.41 | NS | NS | NS | NS | NS | - | NS | 0.45 |
| Zn guts | NS | NS | 0.72 | NS | 0.62 | NS | NS | NS | NS | 0.50 | 0.85 | NS | NS | NS | - | NS |
| Zn liver | NS | NS | NS | 0.73 | -0.39 | 0.40 | NS | 0.64 | NS | NS | NS | 0.75 | NS | 0.45 | NS | - |

**Dam 9:**

Only a significant correlation was found between TL (cm) and weight (Fig. 3S21). There were not found significant (p>0.05) associations among TL and weight with concentrations of the metals in the studied tissues. Instead, the metals levels were significantly correlated between each other (Table 4S9).

**Fig. 3SM21.** Association between TL and weight in tilapias from dam 9 (○ males and ● females) in dam 9.

.

**Table 3SM10**. Spearman correlations (r) among elements concentrations in muscle, liver, gills and guts of *Oreochromis niloticus* at dam 9, significant level p<0.05; NS=not significant (p>0.05).

| Metal  tissue | Cd muscle | Cd  gills | Cd guts | Cd liver | Cu muscle | Cu gills | Cu guts | Cu liver | Pb muscle | Pb gills | Pb guts | Pb liver | Zn muscle | Zn gills | Zn guts | Zn liver |
| --- | --- | --- | --- | --- | --- | --- | --- | --- | --- | --- | --- | --- | --- | --- | --- | --- |
| Cd muscle | - | 0.81 | 0.43 | 0.42 | 0.76 | NS | NS | NS | NS | 0.58 | 0.61 | 0.57 | NS | NS | NS | NS |
| Cd gills | 0.81 | - | 0.53 | 0.51 | 0.68 | 0.45 | NS | 0.44 | NS | 0.65 | 0.50 | 0.59 | NS | NS | NS | NS |
| Cd guts | 0.43 | 0.53 | - | NS | 0.52 | NS | 0.55 | NS | NS | 0.51 | 0.67 | 0.45 | 0.38 | NS | 0.51 | NS |
| Cd liver | 0.42 | 0.51 | NS | - | NS | NS | NS | 0.92 | NS | 0.46 | NS | 0.69 | NS | NS | NS | 0.81 |
| Cu muscle | 0.76 | 0.68 | 0.52 | NS | - | 0.41 | NS | NS | NS | 0.65 | 0.50 | 0.51 | 0.52 | NS | NS | NS |
| Cu gills | NS | 0.45 | NS | NS | 0.41 | - | NS | 0.43 | NS | 0.54 | NS | 0.47 | NS | NS | NS | NS |
| Cu guts | NS | NS | 0.55 | NS | NS | NS | - | NS | NS | NS | NS | NS | NS | NS | NS | NS |
| Cu liver | NS | 0.44 | NS | 0.92 | NS | 0.43 | NS | - | NS | 0.39 | NS | 0.60 | NS | NS | NS | 0.83 |
| Pb muscle | NS | NS | NS | NS | NS | NS | NS | NS | - | NS | NS | NS | 0.38 | -0.43 | NS | NS |
| Pb gills | 0.58 | 0.65 | 0.51 | 0.46 | 0.65 | 0.54 | NS | 0.39 | NS | - | 0.54 | 0.68 | 0.40 | NS | NS | NS |
| Pb guts | 0.61 | 0.50 | 0.67 | NS | 0.50 | NS | NS | NS | NS | 0.54 | - | 0.62 | NS | NS | NS | NS |
| Pb liver | 0.57 | 0.59 | 0.45 | 0.69 | 0.51 | 0.47 | NS | 0.60 | NS | 0.68 | 0.62 | - | NS | NS | NS | 0.44 |
| Zn muscle | NS | NS | 0.38 | NS | 0.52 | NS | NS | NS | 0.38 | 0.40 | NS | NS | - | NS | NS | NS |
| Zn gills | NS | NS | NS | NS | NS | NS | NS | NS | -0.43 | NS | NS | NS | NS | - | NS | NS |
| Zn guts | NS | NS | 0.51 | NS | NS | NS | NS | NS | NS | NS | NS | NS | NS | NS | - | NS |
| Zn liver | NS | NS | NS | 0.81 | NS | NS | NS | 0.83 | NS | NS | NS | 0.44 | NS | NS | NS | - |

**Dam 10**:

A significant correlation was found between TL (cm) and weight (Fig. 3S22). There were not found significant (p>0.05) associations among TL and weight with concentrations of the elements in the studied tissues. Instead the elements levels were significantly correlated between each other (Table 4S10).

**Fig. 3SM22**. Association between TL and weight in organisms from dam 10 (○ males and ● females) in dam 10.

.

**Table 3SM11**. Spearman correlations (r) among elements concentrations in muscle, liver, gills and guts of tilapia at dam 10 significant (p<0.05) and NS = not significant (p>0.05) values.

| Metal  tissue | Cd muscle | Cd  gills | Cd guts | Cd liver | Cu muscle | Cu gills | Cu guts | Cu liver | Pb muscle | Pb gills | Pb guts | Pb liver | Zn muscle | Zn gills | Zn guts | Zn liver |
| --- | --- | --- | --- | --- | --- | --- | --- | --- | --- | --- | --- | --- | --- | --- | --- | --- |
| Cd muscle | - | 0.57 | 0.58 | NS | -0.44 | NS | NS | NS | 0.66 | -0.54 | -0.62 | -0.51 | 0.66 | NS | NS | NS |
| Cd gills | 0.57 | - | 0.47 | NS | -0.52 | NS | NS | -0.47 | NS | NS | -0.51 | -0.45 | NS | NS | NS | NS |
| Cd guts | 0.58 | 0.47 | - | 0.38 | -0.65 | -0.46 | NS | NS | 0.40 | -0.64 | -0.70 | -0.63 | NS | NS | NS | NS |
| Cd liver | NS | NS | 0.38 | - | NS | NS | NS | 0.39 | 0.42 | NS | NS | NS | NS | NS | NS | 0.45 |
| Cu muscle | -0.44 | -0.52 | -0.65 | NS | - | 0.56 | NS | 0.51 | NS | 0.73 | 0.82 | 0.76 | NS | -0.60 | NS | 0.41 |
| Cu gills | NS | NS | -0.46 | NS | 0.56 | - | NS | NS | NS | 0.55 | 0.42 | 0.41 | NS | NS | NS | NS |
| Cu guts | NS | NS | NS | NS | NS | NS | - | NS | NS | NS | NS | NS | NS | NS | NS | NS |
| Cu liver | NS | -0.47 | NS | 0.39 | 0.51 | NS | NS | - | NS | 0.39 | NS | 0.51 | NS | -0.51 | -0.53 | 0.74 |
| Pb muscle | 0.66 | NS | 0.40 | 0.42 | NS | NS | NS | NS | - | NS | NS | NS | 0.51 | NS | NS | NS |
| Pb gills | -0.54 | NS | -0.64 | NS | 0.73 | 0.55 | NS | 0.39 | NS | - | 0.77 | 0.67 | NS | -0.44 | NS | NS |
| Pb guts | -0.62 | -0.51 | -0.70 | NS | 0.82 | 0.42 | NS | NS | NS | 0.77 | - | 0.63 | NS | -0.47 | NS | NS |
| Pb liver | -0.51 | -0.45 | -0.63 | NS | 0.76 | 0.41 | NS | 0.55 | NS | 0.67 | 0.63 | - | NS | -0.51 | -0.45 | 0.57 |
| Zn muscle | 0.66 | NS | NS | NS | NS | NS | NS | NS | 0.51 | NS | NS | NS | - | NS | NS | NS |
| Zn gills | NS | NS | NS | NS | -0.60 | NS | NS | -0.51 | NS | -0.44 | -0.47 | -0.51 | NS | - | 0.39 | NS |
| Zn guts | NS | NS | NS | NS | NS | NS | NS | -0.53 | NS | NS | NS | -0.45 | NS | 0.39 | - | -0.36 |
| Zn liver | NS | NS | NS | 0.45 | 0.41 | NS | NS | 0.74 | NS | NS | NS | 0.57 | NS | NS | -0.36 | - |

**Dam 11:**

A significant correlation was found between TL (cm) and weight (Fig. 3S23). There were not found significant (p>0.05) associations among TL and concentrations of the metals in the studied tissues. Weak correlations were found between weight and Pb (r= 0.40; p=0.04) and Zn (r= 0.42; p=0.03) in muscle. Instead, the metal levels were significantly correlated between each other (Table 4S11)

**Fig. 3SM23**. Association between TL and weight in tilapias from dam 11 (○ males and ● females) in dam 11.

.

**Table 3SM12**. Spearman correlations (r) among elements concentrations in muscle, liver, gills and guts of tilapia at dam 11: significant (p<0.05) and NS = not significant (p>0.05) values.

| Metal  tissue | Cd muscle | Cd  gills | Cd guts | Cd liver | Cu muscle | Cu gills | Cu guts | Cu liver | Pb muscle | Pb gills | Pb guts | Pb liver | Zn muscle | Zn gills | Zn guts | Zn liver |
| --- | --- | --- | --- | --- | --- | --- | --- | --- | --- | --- | --- | --- | --- | --- | --- | --- |
| Cd muscle | - | NS | NS | NS | 0.58 | NS | NS | NS | 0.50 | 0.48 | NS | NS | NS | -0.60 | NS | NS |
| Cd gills | NS | - | 0.43 | NS | NS | NS | NS | NS | NS | NS | NS | NS | NS | NS | NS | NS |
| Cd guts | NS | 0.43 | - | NS | NS | NS | NS | NS | NS | NS | NS | NS | -0.56 | NS | NS | NS |
| Cd liver | NS | NS | NS | - | NS | NS | NS | 0.62 | NS | NS | NS | NS | NS | NS | NS | 0.84 |
| Cu muscle | 0.58 | NS | NS | NS | - | NS | NS | NS | 0.50 | 0.63 | NS | 0.54 | NS | -0.57 | NS | NS |
| Cu gills | NS | NS | NS | NS | NS | - | NS | NS | NS | NS | NS | NS | NS | NS | NS | NS |
| Cu guts | NS | NS | NS | NS | NS | NS | - | NS | NS | NS | 0.40 | NS | NS | NS | NS | NS |
| Cu liver | NS | NS | NS | 0.62 | NS | NS | NS | - | NS | NS | NS | 0.47 | NS | NS | NS | 0.61 |
| Pb muscle | 0.50 | NS | NS | NS | 0.50 | NS | NS | NS | - | NS | NS | NS | NS | NS | NS | NS |
| Pb gills | 0.48 | NS | NS | NS | 0.63 | NS | NS | NS | NS | - | NS | NS | NS | NS | NS | NS |
| Pb guts | NS | NS | NS | NS | NS | NS | 0.40 | NS | NS | NS | - | NS | NS | NS | NS | NS |
| Pb liver | NS | NS | NS | NS | 0.54 | NS | NS | 0.47 | NS | NS | NS | - | NS | NS | NS | 0.41 |
| Zn muscle | NS | NS | -0.56 | NS | NS | NS | NS | NS | NS | NS | NS | NS | - | NS | NS | NS |
| Zn gills | -0.60 | NS | NS | NS | -0.57 | NS | NS | NS | NS | NS | NS | NS | NS | - | NS | NS |
| Zn guts | NS | NS | NS | NS | NS | NS | NS | NS | NS | NS | NS | NS | NS | NS | - | NS |
| Zn liver | NS | NS | NS | 0.84 | NS | NS | NS | 0.61 | NS | NS | NS | 0.41 | NS | NS | NS | - |

**Fig. 3SM24**. Correlation examples between metal concentrations and body size: Cu in muscle vs. TL in dam 1 (A), Pb in muscle vs. TL in dam 2 (B), Pb in guts vs. TL in dam 2 (C), Cu in muscle vs. TL in dam 3 (D), Cu in muscle vs. TL in dam 6 (E), Pb in guts vs. TL in dam 7 (F), Cd in guts vs. TL in dam 7 (G), Pb in gills vs. TL in dam 7 (H), and Cu in gills vs. TL in dam 7 (I). Black and white dots correspond to females and males, respectively.
